# Lumpy skin disease virus suppresses the antiviral response of bovine peripheral blood mononuclear cells that support viral dissemination

**DOI:** 10.1186/s13567-025-01516-w

**Published:** 2025-04-26

**Authors:** Manoj Kumar, Ohad Frid, Asaf Sol, Alexander Rouvinski, Sharon Karniely

**Affiliations:** 1 Department of Microbiology and Molecular Genetics, The Hebrew University-Hadassah Medical School, Institute for Medical Research Israel-Canada, The Hebrew University of Jerusalem, Jerusalem, Israel; 2 Department of Virology, Kimron Veterinary Institute, Beit Dagan, Israel; 3 Department of Parasitology, Kimron Veterinary Institute, Beit Dagan, Israel; 4 The Kuvin Center for the Study of Infectious and Tropical Diseases, The Hebrew University-Hadassah Medical School, The Hebrew University of Jerusalem, Jerusalem, Israel

**Keywords:** MDBK, LSDV, Tropism, PBMCs, ISGs, Recombinant LSDV

## Abstract

Lumpy skin disease virus (LSDV) causes a severe emerging and transboundary disease in cattle. Infection with LSDV leads to the development of widespread dermal nodules. In addition to the skin, LSDV resides in multiple internal organs and can be isolated from the blood of infected cattle. We have characterised the tropism, replication, and dissemination of both a field isolate of LSDV and an attenuated vaccine strain in vitro. To study virus infection and dissemination in living cells, we generated recombinant viruses that express a green fluorescent protein (GFP) under a synthetic viral promoter. The recombinant LSDVs expressing GFP displayed replication kinetics similar to their parental strains in a bovine kidney cell line. These LSDV-GFP strains also replicated effectively in a bovine macrophage cell line and primary bovine foreskin cells, showing no apparent differences between the field isolate and the vaccine strain. Bovine peripheral blood mononuclear cells (PBMCs) infected with either LSDV-GFP strain displayed specific viral-driven GFP fluorescence and significant viral gene expression. However, these infected PBMCs did not support substantial viral DNA replication or the release of infectious progeny. Further analysis of the anti-viral response revealed that heat-treated LSDV, but not infectious viruses, induced the expression of interferon-stimulated genes (ISGs) in PBMCs. Thus, although LSDV did not replicate productively in PBMCs, it evaded the anti-viral response of these cells. Finally, we demonstrated that despite the lack of productive replication, infected PBMCs effectively transmitted LSDV to recipient permissive cells in co-culture, leading to the formation of infection foci. This suggests a potential role for PBMCs in the dissemination of LSDV.

**Supplementary Information:**

The online version contains supplementary material available at 10.1186/s13567-025-01516-w.

## Highlights

- Virulent and attenuated LSDV productively replicated in bovine kidney and bovine macrophage cell lines as well as in primary fibroblasts.
- LSDV showed active viral transcription in PBMCs yet no significant viral genome replication or production of infectious progeny.
- PBMCs infected with heat-treated LSDV but not with fully infectious viruses upregulated ISGs’ RNA.
- PBMCs transmitted and disseminated LSDV to permissive cells by direct contact

**Supplementary Information:**

The online version contains supplementary material available at 10.1186/s13567-025-01516-w.

## Introduction

Lumpy skin disease (LSD) is a severe and emerging transboundary disease that affects cattle, resulting in significant losses for the farming industry [1]. LSD is endemic to many African and Middle Eastern countries and has triggered several outbreaks in Europe over the past decade [2–4]. In recent years, the disease has spread to the Indian subcontinent [5, 6], China [7, 8], and Southeast Asian countries [9], raising significant concerns about its further expansion in developing nations. The World Organization for Animal Health has classified LSD as a notifiable disease [10].

LSD is caused by an enveloped DNA virus of ~150 kb genome from the *Poxviridae* family [11]. The lumpy skin disease virus (LSDV) shows a high degree of genomic similarity to two other members of the *Capripoxvirus* genus: Sheeppox virus (SPPV) and Goatpox virus (GTPV) [12]. These Capripoxviruses share significant antigenic resemblance with LSDV, making them indistinguishable in serological assays. However, LSDV exhibits a high level of host specificity [13, 14].

Despite this strong host specificity in vivo, LSDV demonstrates broad promiscuity in vitro, similar to many other Poxviruses [15]. LSDV can replicate in primary cultured cells and various cell lines derived from cattle [16–18], as well as in cells from different non-permissive host animals, including sheep [18–20], pigs [21], green monkeys [20, 22], hamsters [20, 23], and even humans [24].

In vivo, LSD manifests not only as dermal nodules but can also cause extensive lesions in mucosal, pharyngeal, and gastric membranes, along with inflammation of infected organs [25–27], indicating a broad tissue tropism. LSDV has been isolated from the white blood cells of infected cattle [28, 29].

Transmission of LSDV is primarily thought to occur through blood-feeding arthropods [30–32], although there have been few reports suggesting direct transmission of the virus [33].

Similar to other Poxvirus diseases, the immune control of LSD relies on cell-mediated immunity, which requires vaccination with live attenuated viruses. The LSDV attenuated (Neethling) vaccine was developed through multiple passages of a field isolate in cultured cells and embryonated chicken eggs [34]. This vaccine typically does not cause the fever or skin lesions that are commonly associated with virulent LSDV infection. However, there have been occasional reports of mild vaccine-derived disease [35, 36]. LSDV has developed immunosuppressive strategies to evade the host’s immune response [37], but the specifics of how LSDV interacts with cellular immunity are still poorly understood.

Here, we characterised the cell tropism and replication of both virulent and vaccine LSDV in vitro. We found that both virulent and vaccine LSDV replicated efficiently in a bovine kidney cell line, a bovine macrophage cell line, and in primary bovine foreskin cells. However, bovine peripheral blood mononuclear cells (PBMCs) did not support significant viral DNA replication or the release of infectious progeny, even though they expressed both early and late viral genes.

Additionally, we observed that heat-inactivated LSDVs induced the expression of interferon-stimulated genes (ISGs) in PBMCs, while infectious LSDV suppressed this antiviral activation. Despite the inability of PBMCs to replicate LSDV, these infected cells could transmit the virus to co-cultured MDBK cells, resulting in the formation of infection foci. This finding suggests that PBMCs may play a role in viral dissemination.

## Materials and methods

### Cell lines and viruses

Madin Darby kidney cells (MDBK), obtained from ATCC, CCL-2 were used for viral propagation and recombinant LSDV plaque purification. Bovine skin fibroblasts were prepared from a calf prepuce sourced from a local abattoir. The prepuce tissue was briefly dipped in a povidone-iodine solution, then immersed in phosphate buffer saline (PBS), aseptically minced, and incubated in a trypsin (0.25% w/v) EDTA (0.05% w/v) solution for 15 min. The tissue pieces were allowed to settle, and the supernatant containing detached cells was centrifuged at 200 relative centrifugal force (RCF) for 5 min at room temperature, after which the supernatant was discarded. The cell pellets were resuspended in culture media, as described below and plated in T-25 tissue culture flasks. Foreskin cells were successfully propagated for up to 25 passages.

Both MDBK cells and skin fibroblasts were cultured in minimal essential media (MEM, BI), supplemented with 10% foetal bovine serum (v/v, BI), 1% penicillin–streptomycin-amphotericin B solution (BI), and 1% L-Glutamine (v/v, BI) in a 5% CO_2_ humidified incubator at 37 °C. Immortalised peritoneal bovine macrophage cells (BoMacs), generously provided by Judith Stabel (USDA, [38]), were cultured in RPMI-1640 media (BI), supplemented with 10% foetal bovine serum, 1% antibiotics, and 1% L-Glutamine (v/v, BI) in a 5% CO_2_ humidified incubator at 37 °C.

### Formation of MDBK-Ruby cells

MDBK cells that stably express a cytoplasmic red fluorescent protein, mRuby-3, under the EF-1 alpha core promoter (MDBK-Ruby) were generated through lentivirus transduction. These MDBK-Ruby cells produce bicistronic mRNA, which encodes mRuby-3, followed by a 2A self-cleaving peptide and a puromycin resistance enzyme (PAC) coding sequence. To select for transgene integration, MDBK-Ruby cells were treated with 2 µg/mL puromycin for 48 h. After this initial selection, the cells were split at a ratio of 1:4 and continued to be selected with puromycin for an additional 48 h until all control non-transduced cells were eliminated. The puromycin-selected cells were confirmed to express Ruby by fluorescence microscopy.

### LSDV propagation

Virulent LSDV was isolated from a skin nodule of infected cattle during the 2006 LSD outbreak in Israel (cell culture passage number 3). This LSDV field isolate and the Vaccine strain (OBP strain, batch no. 449, Onderstepoort Biological Products SOC Ltd) were used in the experiments and for genetic manipulation.

These strains were propagated in MDBK cells as follows: MDBK cells were inoculated with the virus for 1 h, washed with PBS, and refreshed with media. The cells were cultured for 8 to 10 days to allow several rounds of viral replication. They were monitored daily for developing a cytopathic effect (CPE) until the monolayer was almost completely disrupted.

Supernatants were collected and subjected to three freeze–thaw cycles to properly release the virus. They were then centrifuged at 300 RCF for 5 min at room temperature to eliminate debris and stored in aliquots at −80 °C. The LSDV strains were heat treated (HT) by incubating the viral preparations at 55 °C for 30 min in a water bath.

### Viral titre determination

MDBK cells were used to determine LSDV titre. Tenfold dilutions of individual strains (with at least six repeats for a single strain) were inoculated into MDBK cells that had already been pre-seeded in 96-well plates. After 4 to 6 days, the cells were examined for the development of CPE, and the endpoint dilution was recorded. The TCID_50_ was calculated using the Reed and Muench method [39].

### Reagents

Minimal essential media, RPMI-1640 media, fetal bovine serum, penicillin–streptomycin-amphotericin B solution, L-Glutamine, and DPBS were procured from Biological Industries (BI). Actinomycin D, carboxymethyl-cellulose (CMC), cycloheximide (CHX), puromycin, and phosphonoacetic acid (PAA) were obtained from Sigma Aldrich. The pGMEM-T Teasy kit (Promega), Lipofectamine 2000 reagent (Invitrogen, USA), the Gibson assembly cloning kit (NEB), Ficoll-paque plus solution (GE Healthcare), the RNeasy kit (Qiagen), the DNeasy kit (Qiagen), the Dream Taq PCR master mix (Thermo Scientific), qPCR bio SyGreen blue mix (PCR biosystems), the Turbo DNA free kit (Invitrogen), the SensiFAST cDNA synthesis kit (Bioline), propidium iodide (PCR biosystems), BOVIGAM pokeweed mitogen (Applied Biosystems) were also used.

### Construction of LSDV-GFP recombination plasmid

LSDV expressing GFP was developed by modifying previously established strategies [40, 41]. In summary, a recombination plasmid was created from a linear pGME-T vector (Promega) by fusing it with a cassette that contained left and right flanking arms, both featuring LSDV target sequences, along with a GFP sequence under a synthetic Vaccinia early/late promoter [40, 42] (Additional file 1). The intergenic region between LSDV05 (left arm) and LSDV06 (right arm), which maintains the end-to-end orientation of ORFs, was selected as the genomic insertion target to minimise potential interference with viral gene expression.

The flanking arms and GFP sequence were amplified from viral DNA extracted from LSDV-infected MDBK cells and pEGFPN-1 (Clontech) plasmid, respectively. PCR was conducted using Phusion high-fidelity DNA polymerase (NEB) with sets of primers designed to introduce homology regions corresponding to LSDV genes and homology sequences compatible with pGME-T for Gibson cloning (Additional file 2). Each PCR fragment was separated using agarose gel electrophoresis and purified with the GeneAll Expin kit (GeneAll Biotech Ltd). Following the manufacturer’s instructions, the purified PCR fragments were then joined with the pGEM-T plasmid through Gibson assembly cloning (NEB). Sanger sequencing was performed to confirm no changes in the sequence of the GFP recombination cassette.

### Construction and purification of LSDV–GFP recombinant viruses

Confluent MDBK cells in 6-well plates were infected with either field or vaccine strains of LSDV at MOI = 0.5. After one hour of incubation, the inoculum was removed, and the cells were washed twice with PBS. The cells were replenished with 1 mL of MEM media containing 5% FBS.

Next, 10 μL of Lipofectamine 2000 reagent (Invitrogen) was complexed with 5 μg of the pGEM-T-LSDV-GFP plasmid in 300 μL Opti-MEM (Invitrogen) and gently added to the wells. The culture plate was subsequently placed in an incubator, allowing for viral replication and recombination events that produced GFP-expressing LSDV. The following day, the cells were gently washed with PBS and replenished with fresh MEM media.

Cells were monitored for up to 50 h or until they exhibited CPE and the appearance of green fluorescent foci. It is important to note that cells transfected with the pGEM-T-LSDV-GFP plasmid without prior LSDV infection showed no green fluorescence, confirming the stringent control of the synthetic Vaccinia early/late promoter.

The media were then collected for plaque purification of GFP-expressing recombinant LSDV viruses. Media harvested from the transfected cells were inoculated onto confluent MDBK cells for one hour, washed three times with PBS to remove the inoculum and overlaid with 1.5 mL of CMC solution (0.5% CMC (w:v), 10% MEM (v/v), 0.0025% Na_2_HCO_3_ (v/v), 10% foetal bovine serum (v/v), 1% antibiotics, 1% L-glutamine (v/v), and 30% sterile water). The plate was then incubated in a CO_2_ incubator.

After 24–48 h, one of the distinct fluorescent foci seen under the microscope was selected using a micropipette tip (avoiding non-fluorescent foci) and submerged in media for another round of purification. This process was repeated for a total of three rounds to obtain pure recombinant LSDV. The purity of the recombinant LSDV (from parental viruses) was confirmed using PCR.

DNA extracted from the recombinant LSDV stocks (using the DNeasy kit, Qiagen) was used for PCR with three sets of primers (Additional file 2). PCR was performed using a 2xDream Taq PCR master mix (Thermo Scientific) according to the manufacturer’s instructions. In brief, a 20 µL reaction was prepared with 10 µL PCR mix (2x), 1 µL (set A, Additional file 2) or 0.5 µL (sets B and C, Additional file 2) of 10 µM forward and reverse primers, 3 µL template DNA, and 5 µL distilled water. The thermal cycler program included 95 °C for 1 min, 35 cycles of: 95 °C for 15 s, 58 °C (set A, Additional file 2) or 60 °C (sets B and C, Additional file 2) for 30 s, and 72 ºC for 85 s.

### PBMCs isolation and culture

Peripheral blood was collected from the tail vein of Holstein calves using Becton Dickinson (BD) vacutainer heparin tubes. The procedure for bleeding the calves to prepare PBMCs was approved by the Kimron Veterinary Institute Committee on Animal Research and Ethics (Ref. No. 102–2023).

We used density gradient centrifugation with a Ficoll-paque layer (density 1.086 g/mL, GE Healthcare) to separate blood cells, following slight modifications to the manufacturer’s instructions. Blood was diluted in a 1:1 ratio with PBS containing 2% fetal bovine serum. This mixture was layered on top of Ficoll-paque in SepMate tubes (Stem cell technologies) and centrifuged at 1200 RCF for 10 min at room temperature. In the SepMate tubes, PBMCs formed a whitish layer.

The PBMCs layer was carefully and immediately decanted into a new tube for re-centrifugation at 600 RCF for 8 min at room temperature. The PBMCs were washed with PBS and pelleted again at 600 RCF for 8 min at room temperature. The harvested PBMCs were suspended in RPMI-1640 medium supplemented with 10% FBS, 1% L-glutamine, and 1% antibiotics. Viable cells were counted using the trypan blue dye exclusion method, seeded in culture plates and incubated overnight in a 5% CO_2_ humidified incubator at 37 °C before being used for LSDV infection.

### Flow cytometry analysis

MDBK, BoMac, and bovine foreskin cells were collected using trypsinisation. PBMCs were gently scraped with slow stirring in PBS. The collected cells were fixed at room temperature for 10 min using 4% neutral buffered formalin, then passed through a 70-micron cell restrainer and processed in a FACS analyser (BD FACS Calibur, Model E4856,). A total of 20 000 events were recorded for each sample.

### Quantification of PBMCs viability

The viability of PBMCs was assessed using propidium iodide (PI) exclusion. In brief, 10% (v:v) of a PI stock solution (20 μg/mL in PBS) was added to uninfected and LSDV-infected PBMCs for 10 min. Afterwards, the cells were supplemented with PBS for FACS analysis. The percentage of viable cells was calculated by subtracting the percentage of PI-positive cells from 100%.

### LSDV infection of PBMCs

PBMCs were seeded in 12-well plates at a density of 0.5 million cells per well. The following day, the cells were inoculated with either recombinant or parental strains of LSDV. After gentle washing with PBS, the cells were supplemented with complete RPMI-1640 media. In some cases, the viral DNA polymerase inhibitor PAA and the translation inhibitor CHX were added. Cells were lysed at specified time points to extract DNA and RNA for the quantitative analysis of DNA replication and gene expression. Additionally, PBMCs were stimulated by overnight incubation with pokeweed mitogen at a concentration of 5 µg/mL/million cells to monitor LSDV replication. The activity of pokeweed mitogen was confirmed by measuring the release of IFN-γ into the medium of treated PBMCs using a commercial IDscreen ruminant IFN-γ ELISA kit (IDvet).

### DNA /RNA extraction, reverse transcription (RT), and quantitative PCR (qPCR)

DNA and RNA extraction and purification from cells were performed using the Qiagen RNeasy and DNeasy Blood and Tissue kits. The total RNA of all samples was quantified with a Nanodrop reader (Thermo Scientific), DNA was then removed using the Turbo DNA free kit (Invitrogen), and cDNA was synthesised using the SensiFAST cDNA synthesis kit (Bioline) according to the manufacturer’s instructions.

For the qPCR reaction, a total volume of 10 µL was prepared, consisting of 5 µL of 2 × qPCR bio SyGreen blue mix (PCR biosystems), 1 µL of forward primer (10µM), 1 µL of reverse primer (10µM), and 3 µL of diluted cDNA. The qPCR reactions were conducted on a MIC real-time PCR cycler (Bio Molecular Systems, BMS) using the following program: pre-denaturation at 95 °C for 3 min, followed by 45 cycles of 95 °C for 15 s, 55 °C for 15 s, and 60 °C for 25 s.

Samples that did not undergo DNA digestion were assessed for LSDV genome replication. The relative expression of viral or host genes was calculated using the delta-delta CT method, expressed as fold change in histograms, with GAPDH as the reference gene. The time point after virus inoculation (for viral genes and GFP expression) or mock infection (for ISG expression) was used as the control condition.

### LSDV dissemination assay

PBMCs were infected with either LSDV WT-GFP or LSDV Vac-GFP for two hours. Following the inoculation, the PBMCs were washed once with PBS, and media was added to gently scrape the cells from the well by stirring. The PBMCs were then pelleted at ~600 RCF for 1 min, washed twice with PBS resuspended in MEM complete media, and poured over pre-seeded confluent MDBK or MDBKRuby cells.

After allowing the PBMCs to settle for 2 h (confirmed through microscopic observation), the media was removed, and a 0.2% agarose overlay (in complete MEM media) was applied. Co-culture experiments using primary bovine fibroblasts as donor cells were conducted in a similar manner, with fibroblasts collected after infection by trypsinisation. The emergence of foci was monitored by fluorescence microscopy, and images were captured.

After 48 or 96 h, the cells were fixed with 4% neutral buffered formalin and counter-stained with Hoechst dye (1 µg/mL). Plate imaging was performed using the Cytation microplate imager, version 5 (Biotek), utilising the GFP channel (Ex 470 nm/ Em 525 nm) for the detection of fluorescent green cells foci and the DAPI channel (Ex 377 nm/ Em 454 nm) for the detection of all cells (nuclear stained). Image analysis was conducted using the Cytation Gen5 software with a threshold circumference of 600 µm ± 50 for counting the fluorescent cell foci.

### Transwell experiments

MDBK cells were seeded in 12 mm diameter insets containing cell culture-treated polycarbonate membranes with a pore size of 0.4 µm (Corning). The following day, PBMCs pre-seeded in a 12-well plate were infected with either LSDV WT-GFP or LSDV Vac-GFP for two hours. After the inoculation, the PBMCs were washed twice with PBS and once with RPMI complete media before being replenished with RPMI complete media. The insets containing MDBK cells were then gently transferred into the wells with infected PBMCs for co-incubation. Additionally, freshly trypsinised MDBK cells were seeded onto the infected PBMCs in control wells for co-culturing.

### Fluorescence microscopy

Mock-infected**/**LSDV-infected cells were imaged using a fluorescence microscope (Nikon T-DH, Japan) with an LED *p*E excitation system (CoolLED, UK).

### Fluorometric determination of preformed GFP

LSDV/ LSDV-GFP stocks (prepared in MDBK cells) were quantified for GFP presence in the media using a fluorometric approach with the DeNovix DS-11 (Thermo Fisher). In brief, 250 µL of media from LSDV GFP stocks were analysed by exciting the samples at 488 nm and measuring the emission at 506 nm. Media from uninfected cells and LSDV stocks were used to check background fluorescence and normalise the RFU values.

### Statistical analysis

GraphPad Prism 9.1.0 software was used for statistical analysis and plotting the graphs. Data values were presented as mean ± standard error of the mean (SEM). The statistical tests used to analyse experimental data are indicated in the figure legends.

## Results

### Recombinant LSD viruses expressing GFP productively replicate in cultured bovine cells

To facilitate the monitoring of LSDV infection and dissemination through flow cytometry and fluorescence microscopy, we have constructed recombinant versions of both virulent and vaccine-attenuated LSDV that express a GFP reporter gene. These are referred to as LSDV WT-GFP and LSDV Vac-GFP, respectively. The GFP gene was cloned under a synthetic early/late promoter from the Vaccinia virus [42] and targeted to the viral genome between *LSDV05* and *LSDV06* genes using homologous recombination, ensuring that the flanking genes were not disrupted (Additional file 1).

MDBK cells were infected with the WT or Vac LSDV strains and transfected with a plasmid containing the recombination cassette. Recombinant viruses were isolated through three rounds of plaque purification from the green-fluorescent foci of the infected cells (Additional file 1A). The homogeneity of these isolated recombinant viruses was confirmed through PCR (Additional file 1B).

**Figure 1:**
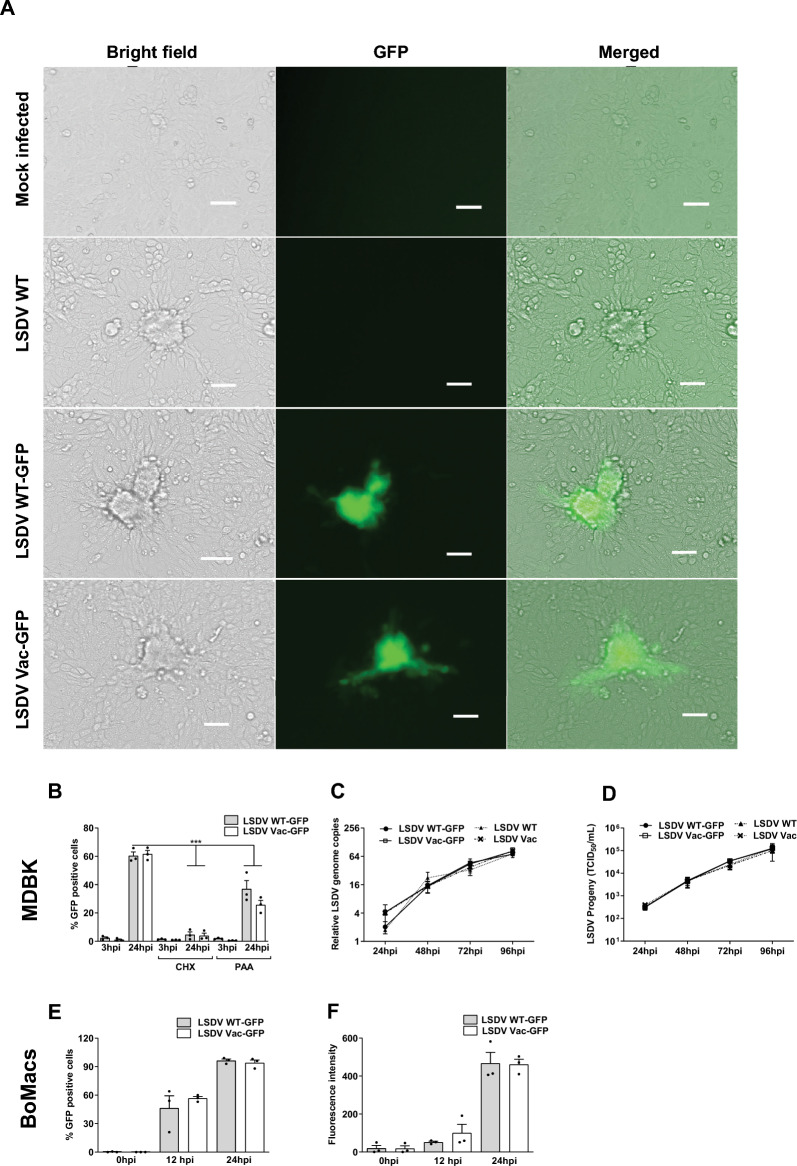
**WT and Vaccine LSDV GFP strains efficiently replicate in MDBK and BoMac cells.** (**A**) MDBK cells were inoculated with parental LSDV and recombinant LSDV GFP strains at MOI = 3. After 48 h, cells were imaged using epifluorescence microscopy to monitor the morphology and fluorescence of infection foci (Scale Bar 100 µm). **(B)** MDBK cells were inoculated with LSDV WT-GFP and LSDV Vac-GFP at MOI = 1, where indicated cells were pretreated with CHX or the viral DNA polymerase inhibitor PAA. Cells were collected at the time points indicated, and the percentage of GFP-positive cells was determined using flow cytometry. **(C-D)** MDBK cells were infected with parental or LSDV- GFP viruses at MOI = 3 for 1 h. At 1, 24, 48, 72, and 96 hpi, cells were harvested and assayed for relative viral DNA quantification by qPCR (**C**) and infectious progeny by TCID_50_ titration (**D**). **(E–F)** BoMac cells were infected with LSDV WT-GFP and LSDV VAC-GFP at MOI = 3 for 1h (T = 0). At the time points indicated, infected cells were collected and analysed by flow cytometry. The percentage of GFP-expressing cells (**E**) and the mean fluorescence intensity were determined (**F**). Data values were expressed as mean ± SEM representing three biological replicates in the above graphs (**B**-**F**).

When MDBK cells were infected with LSDV WT-GFP or LSDV Vac-GFP, the percentage of GFP-expressing cells (Figure 1B and Additional files 3A-C) increased over time, as did the intensity of fluorescence signal per cell (Additional file 3D). This increase corresponded with the progression of recombinant virus replication in the host cells. Notably, GFP accumulation did not occur when the LSDV-GFP infected MDBK cells were pre-treated with the translation inhibitor cycloheximide (CHX) (Figure 1B), confirming that GFP is synthesised de-novo in the infected cells. Furthermore, the percentage of GFP-positive cells significantly decreased when cells were treated with phosphonoacetic acid (PAA, Figure 1B), a known inhibitor of DNA replication in Herpes and Pox viruses [43]. This finding supports the conclusion that GFP expression is dependent on viral replication.

A time-course infection of MDBK cells demonstrated that the kinetics of viral DNA replication (Figure 1C) and the production of infectious virus progeny (Figure 1D) were similar between the parental LSDVs and the GFP recombinant LSDVs. This indicates that the integration of GFP did not hinder viral replication. The replication kinetics were also comparable between WT and Vac LSDVs.

In vivo, the highest concentrations of LSDV are found, and it can be most easily isolated from skin nodules of infected cattle [44–46]. For this reason, we opted to use primary fibroblast cells derived from the prepuce of a calf to characterise recombinant LSDV strains further. Similar to MDBK cells, primary fibroblasts infected with LSDV WT-GFP or LSDV Vac-GFP exhibited increasing GFP fluorescence over time (Additional files 3E-H).

Previous histopathological and immunohistochemical analyses of tissues from LSDV-infected cattle have detected typical Poxvirus inclusion bodies [27] and LSDV antigens [25, 47] within macrophages in the skin and lymph nodes. We found that both virulent and attenuated LSDV could infect and replicate in immortalised peritoneal bovine macrophage cells (BoMac cells, [38]) with similar efficiencies (Figures 1E and F). However, it is essential to note that these transformed monocytic (BoMac) cells have lost some functional properties [48]. Consequently, we also tested LSDV replication and the host cell’s innate immune response to the infection in primary peripheral blood mononuclear cells.

### Bovine PBMCs are susceptible to LSDV infection yet do not support significant viral replication

Preparations of buffy coats from cattle blood, whether naturally or experimentally infected with LSDV, have been shown to contain viral DNA and infectious virus [26, 28, 29]. However, active viral replication in these cells has not been confirmed.

We isolated PBMCs from naïve calves that had neither been previously exposed to LSDV nor vaccinated against it. After seeding the cells in culture plates and incubating them overnight, we infected them with LSDV WT-GFP and LSDV Vac-GFP at MOI = 3. This MOI was determined based on the titration of virus stocks on MDBK cells.

**Figure 2:**
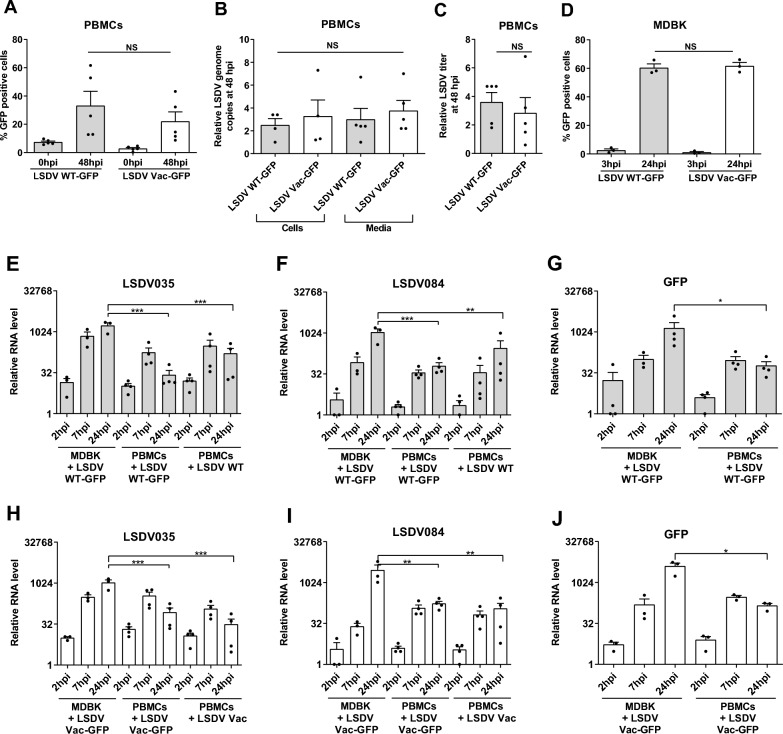
**PBMCs are susceptible to LSDV-GFP (WT and Vac) infections but poorly support viral replication.** (**A-C**) PBMCs were inoculated with LSDV-GFP strains at MOI = 3 for 2 h (T = 0) and further incubated for 48 h. At both time points, cells were collected for flow cytometry analysis (**A**), DNA extracted from infected PBMCs (B, left bars) and collected media (B, right bars) were assayed for relative genome copy numbers. Medium was also assessed for the relative increase in virus progeny titre (TCID_50_) (**C**). Titration involved half-log dilution to allow better resolution of titres. **(D)** MDBK cells were inoculated with LSDV-GFP strains parallel to PBMCs as described above (at MOI = 1) and analysed for the percentage of GFP-positive cells by flow cytometry. **(E-J)** MDBK cells or PBMCs were inoculated with parental LSDV and recombinant LSDV GFP strains, as indicated, at MOI = 1 for 1 h. Relative changes in the levels of transcripts encoding the reporter gene GFP and viral genes (LSDV035 and LSDV84) at the indicated time points were determined by RT-qPCR. Three independent sets were used to plot the figures and data values presented as mean ± SEM. One-way ANOVA followed by Tukey’s post-hoc test was used to compare all groups (E–F, H-I). Ratio paired t-test was used in G and J. ∗ *p* < 0.05, ∗∗ *p* < 0.01, ∗∗∗ *p* < 0.001, NS- non-significant.

After 2 h of inoculation (T = 0), ~5% of the PBMCs became GFP-positive. This percentage increased to around 25% after 48 h, suggesting an effective MOI in PBMCs of 0.25. This indicates that PMBCs are less susceptible to infection than MDBK cells (Figure 2A). Notably, we observed significant variation in the percentages of GFP-positive cells across different independent preparations of PBMCs (Figure 2A), unlike the low variation observed when infecting homogenous populations of bovine cell lines or primary fibroblasts (Figures 1B, E and Additional files 3C, G).

Both LSDV WT-GFP and LSDV Vac-GFP were found to be equally infectious to adherent PBMCs (Figure 2A). The intensity of the GFP signal in the infected PBMCs did not significantly increase over the course of infection (Additional file 4A). In contrast, a dramatic increase was observed in the infected MDBK, BoMac cell lines, and primary fibroblasts (Additional file 3D, Additional file 3H and Figure 1F, respectively). Furthermore, the number of viral DNA copies in LSDV-infected PBMCs did not show a significant increase (Figure 2B, left bars). The media collected from infected PBMCs indicated only a minor increase (*ca* threefold) in viral DNA (Figure 2B, right bars) or in the production of infectious viral progeny (Figure 2C) during PBMCs infection with either LSDV WT-GFP or LSDV Vac-GFP.

We also monitored viral replication in pokeweed mitogen-stimulated PBMCs, as cellular proliferation may support viral replication, as has been shown for other viruses [49, 50]. However, we found only a minor increase in LSDV DNA copies during infection, similar to what was observed in non-stimulated PBMCs (Additional file 4B).

We next investigated whether the GFP signal observed in infected PBMCs was due to carryover of preformed GFP from LSDV-GFP viral stocks produced in MDBK cells rather than resulting from infection-induced GFP expression in PBMCs. To address this, we conducted two control experiments: 1) We inoculated PBMCs with heat-treated (HT) LSDV viruses (Additional file 5A). 2) We arrested cellular translation in PBMCs using CHX before infection with LSDV-GFP viruses (Additional file 5B).

Both treatments successfully prevented the appearance of green fluorescence, which was otherwise observed in untreated PBMCs inoculated with infectious viruses. This confirms that GFP is synthesised de-novo in LSDV-GFP-infected PBMCs.

It is worth noting that GFP exhibits high thermal stability [51]. We confirmed that the heat treatment (30 min at 55 °C), which resulted in a 100-fold reduction in virus titres (Additional file 5C), did not affect the fluorescence of any preformed GFP found in the LSDV-GFP stocks (Additional file 5D).

Next, we examined whether the inability of PBMCs to support LSDV replication productively was related to virus-induced cell death. As determined by PI exclusion, there was no significant difference in viability between uninfected PBMCs and those infected with either LSDV WT-GFP or LSDV Vac-GFP. All tested groups maintained over 90% viability (Additional file 6).

### LSDV exhibits progressive viral gene expression in infected PBMCs

We further investigated whether LSDV infection of PBMCs is associated with a failure to execute its transcriptional program. To explore this, we selected four LSDV genes *(LSDV035*, *LSDV076*, *LSDV084* and *LSDV089*) to track viral transcription in infected cells. Given the limited understanding of the regulation of LSDV gene expression [52], we based our choice of viral genes on their homology to the Vaccinia virus [11], aiming to represent both early and late viral genes (Additional file 2).

Initially, we characterised the expression of these viral genes and the reporter GFP gene during productive LSDV-GFP replication in MDBK cells and fibroblasts (Figures. 2E-J, Additional files 8A-I). All four LSDV genes and the GFP exhibited an increase in abundance as early as 2 hpi, with a similar fold increase at 24 hpi. Specifically, *LSDV035* (Figures 2E, H and Additional file 8E) and *LSDV076* (Additional files 8A, C and F) were found to be relatively more abundant than the other LSDV genes and the GFP at the early time points of 2 hpi and 7 hpi in both MDBK and fibroblasts.

The dependency of late viral gene expression on viral DNA replication has been previously demonstrated for the Vaccinia virus [53, 54]. The gene *LSDV084* met the criteria for a late gene, as its expression was inhibited by PAA treatment, which interferes with viral genome replication (Additional file 7A). Conversely, *LSDV035* remained unaffected by PAA, supporting its classification as an early LSDV gene (Additional file 7B). The expression patterns of all tested viral genes did not show significant differences between WT and Vac LSDV.

MDBK cells infected with heat-treated LSDV demonstrated a lower induction of both viral genes and GFP (Additional file 9). The incomplete inhibition of viral transcription may be attributed to the insufficient inactivation of the viruses using the heat procedure we employed (Additional file 5C).

We then examined the expression of LSDV genes in infected PBMCs. Our findings showed that the RNA levels of all selected viral genes (*LSDV035*, *076*, *084*, *089*), as well as GFP, increased in abundance in PMBCs at both 7 hpi and at 24 hpi (Figures 2E-J and Additional files 8A-D). When HT viruses were used to infect PBMCs, the RNA levels of both viral and GFP genes were significantly reduced (Additional file 10).

LSDV-infected PBMCs showed only a slight decrease in viral transcript levels up to 24 hpi when treated with CHX, indicating that protein synthesis is unnecessary for their transcription. This transcription may be driven by pre-existing proteins (Additional file 11). The induction levels of viral genes in PBMCs infected with LSDV-GFP reached approximately a 100-fold increase at 24 hpi (Figures 2E-J). However, this increase was tenfold lower than the more than 1000-fold increase observed in MDBK cells at the same time point (Figure 2E-J).

PMBCs infected with the parental (non-recombinant) LSDV exhibited a similar expression pattern to that of LSDV-GFP viruses (Figures 2E-J and Additional files 8A-D). While LSDV remains transcriptionally active in PBMCs, it does not achieve significant productive replication.

### Infectious LSDV suppresses ISG induction in PBMCs

**Figure 3:**
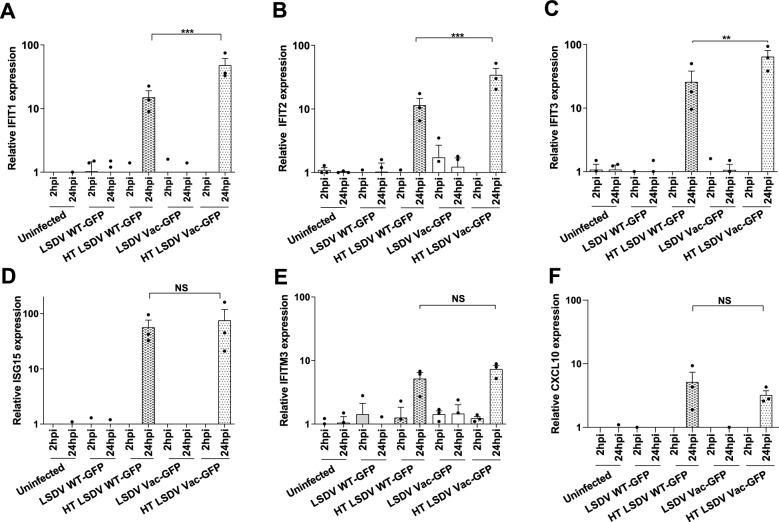
**Heat-treated but not infectious LSDV strains upregulate ISGs and CXCl10 transcripts in PBMCs.** (**A-F**) A comparative analysis of ISGs (IFIT1,2,3, ISG15 and IFITM3) expression performed in uninfected PBMCs or PBMCs inoculated with infectious LSDV-GFP and HT LSDV-GFP viruses at MOI = 1. RT-qPCR monitored relative changes in the levels of transcripts encoding ISGs at the indicated time points. The relative expression of viral genes for this experiment is shown in Additional file 10. Fold changes were plotted in bar graphs as mean ± SEM for three independent biological sets. One-way ANOVA was used to compare all means, and post-hoc Tukey’s test was followed to test the significance level. ∗  ∗ *p* < 0.01, ∗  ∗  ∗ *p* < 0.001, NS- non-significant.

Previous research has demonstrated that an intact type I interferon response limits productive replication of Poxvirus [55]. We aimed to investigate whether the nonproductive replication of LSDV in PBMCs is linked to a cellular anti-viral response. To achieve this, we monitored the kinetics of expression of ISGs (*IFIT1*, *IFIT2*, *IFIT3*, *ISG15* and *IFITM3*) in PBMCs inoculated with both virulent and attenuated strains of LSDV, along with their HT preparations (Figures 3A-E).

All tested ISGs showed a significant increase in expression when exposed to the HT viruses, but not with infectious LSDV. Specifically, *IFITM3* RNA levels experienced a moderate increase—about tenfold—while the other ISGs were induced approximately 100-fold. Notably, IFIT1-3 were induced to higher levels by HT Vac-LSDV compared to HT WT-LSDV. In MDBK and BoMac cells, which support productive LSDV replication, neither the infectious nor the HT viruses significantly affected ISG RNA levels Additional file 12).

IFNγ is a crucial immune stimulatory cytokine secreted by various immune cells in response to viral infection [56]. Our research indicates that HT LSDV, but not infectious viruses, triggers the expression of the IFNγ induced chemokine gene CXCL10 (Figure 3F) in PBMCs at 24 hpi. Similar levels of CXCL10 induction were observed with both HT WT and HT Vac LSDV. In summary, our findings suggest that infectious LSDV suppresses both type I and Type II interferon responses.

### Infected PBMCs can disseminate LSDV to neighbouring permissive cells

Our results demonstrate that LSDV can infect adherent PBMCs; however, its replication within these cells appears negligible. We wanted to investigate whether LSDV-infected PBMCs could transmit the virus to neighbouring susceptible cells. To explore viral dissemination, we first infected PBMCs with either the WT or Vac LSDV-GFP for 1 h. After extensive washing, we seeded these cells onto a monolayer of MDBK cells, which were overlaid with semi-solid media. The cultures were incubated at 37 °C and monitored for over a 48 h period.

**Figure 4:**
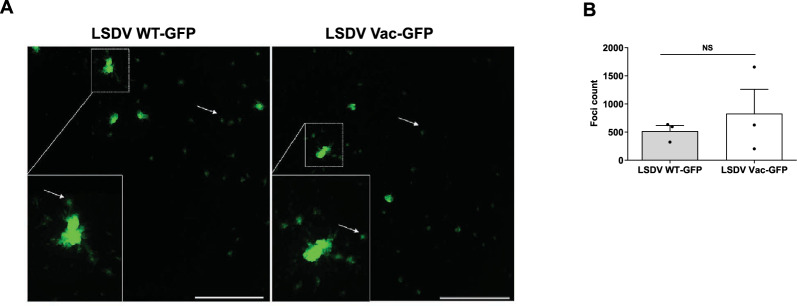
**WT and Vac LSDV GFP viruses disseminated to MDBK cells from infected PBMCs.** (**A-B**) PBMCs were infected with WT and Vac LSDV-GFP at MOI = 1 for 2 h, extensively washed, collected, and seeded on a monolayer of MDBK cells in a 6-well plate, overlaid with semi-solid media and incubated at 37 °C for 48 h. Fluorescent foci per well (**A**), representative images, scale bar 1000 µm) were counted using a Cytation microplate reader (**B**). Three independent repeats were used to plot the histogram, and values were presented as mean ± SEM. Paired t-test was used for statistical analysis. NS- non-significant.

Single green fluorescent MDBK cells were observed as early as 12 h after co-culturing with infected PBMCs, and discrete fluorescent cell foci had formed by 24 h (data not shown), indicating the transfer of infectious LSDV from PBMCs to MDBK cells. The size of these fluorescent foci in the co-cultured MDBK cells increased from 24 to 48 h (Figure 4A). We found no significant difference in the ability of WT and Vac LSDV-GFP strains to disseminate from PBMCs to MDBK cells, as shown by the foci counts (Figure 4B).

To distinctly mark recipient-infected MDBK cells, we repeated the co-culture experiments using MDBK-Ruby cells, which stably express the red fluorescent protein mRuby3 (Materials and methods). Foci of recipient cells expressing both GFP and Ruby were clearly visible when MDBK-Ruby cells were co-cultured with LSDV-GFP infected PBMCs (Additional file 13A), confirming that PBMCs facilitate the dissemination of LSDV to MDBK cells. Additionally, infected bovine fibroblasts were also capable of spreading LSDV to co-cultured MDBK-Ruby cells (Additional file 13B).

To differentiate between indirect and direct cell-to-cell transmission of LSDV from infected PMBCs, we used transwells to physically separate the donor-infected PBMCs from the recipient MDBK cells (Additional file 13C). MDBK cells were placed in transwell inserts with a 0.4 µm pore size membrane (which allows virions but not cells to pass through) and positioned into wells pre-seeded with LSDV-GFP infected PBMCs that had been extensively washed after inoculation. In parallel, control wells contained infected PBMCs directly overlaid with MDBK cells.

After 4 days, only a few infected foci appeared in the transwells, accounting for < 0.001% of the recipient MDBK cells (Additional file 13C, left). In contrast, ~20% of MDBK cells were infected in the directly overlaid co-cultures at 2 dpi (not shown), increasing to over 90% at 4 dpi (Additional file 13C, right).

In summary, while WT and Vac LSDV poorly replicate in PBMCs, they effectively utilise PBMCs to disseminate to neighbouring permissive cells through direct contact-dependent cell-to-cell transmission.

## Discussion

Fever, ocular and nasal discharge, and the development of skin nodules are typically associated with virulent LSDV infection in cattle. However, these clinical signs are rare and mostly absent in cattle vaccinated with the attenuated Neethling strain [35, 36]. The pathogenesis of both virulent and attenuated LSDV may involve differences in cell and tissue tropism, replication, and dissemination properties.

We have characterised the replication of LSDV in bovine cultured cells using recombinant GFP reporter versions of both virulent (WT) and vaccine-attenuated viruses (Figure. 1, Additional file 1). LSDV WT-GFP and LSDV Vac-GFP exhibited similar replication kinetics compared to their parental strains and one another in MDBK cells (Figure. 1C-D), aligning with previous studies conducted in these cells [17]. Additionally, LSDV WT-GFP and LSDV Vac-GFP replicated with comparable efficiency in primary bovine foreskin cells (Additional files 3E-H).

In vivo, skin nodules do not typically form in most vaccinated cows. When skin nodules do appear in a minority of LSD-vaccinated cows, they are smaller and contain less virus than those found in cattle infected with the virulent strain [35, 36]. Our in vitro findings suggest that this phenotypic difference is not due to a reduced ability of the attenuated LSDV to target skin cells. Similarly, we observed no differences in the replication of LSDV WT-GFP and LSDV Vac-GFP in immortalised peritoneal bovine macrophage cells (Figures 1E-F), even though in vivo, only the virulent LSDV strain was widely detected in lymph nodes and whole blood, where macrophages can be found [45].

Preparations of buffy coats from the blood of both naturally [29] and experimentally infected cattle [26, 28, 45] contain LSDV DNA as well as infectious virus particles. When introduced through biting insects, LSDV may encounter circulating bovine blood cells. Interestingly, intravenous injection of the virus resulted in a more consistent and widespread disease than intradermal inoculation in experimental conditions [28].

In experiments involving the mechanical transmission of LSDV by biting insects, LSDV DNA was detected in the blood of recipient cattle either before or at the same time as the appearance of skin nodules. However, viremia under these experimental conditions peaked several days later [31, 57, 58]. Despite these findings, it was unclear whether LSDV actively replicates in blood cells.

We found that adherent PBMCs isolated from naïve cattle were susceptible to infection with LSDV WT-GFP and LSDV Vac-GFP, as indicated by the appearance of GFP (Figure 2A). However, PBMCs did not support productive LSDV replication, as evidenced by a minimal increase in viral DNA within the cells and negligible secretion of infectious progeny into the medium (Figures 2B and C). The limited ability of LSDV to replicate in PBMCs was not related to virus-induced cell death (Additional file 6). It was not enhanced by stimulating PBMC with pokeweed mitogen (Additional file 4B).

What could explain the poor replication of LDV in PBMCs compared to other cultured bovine cells? The availability of receptors on host cells plays a crucial role in determining the tropism of many viruses. However, specific cellular receptors for LSDV are currently unknown. Recent studies have shown that LSDV can utilise a primarily receptor-independent pathway of macropinocytosis to enter host cells [59].

In general, the cellular tropism of Poxvirus is believed to be limited by post-entry events rather than the availability of receptors [15]. For instance, subsets of 3T3 murine fibroblasts that were non-permissive to Myxoma virus replication exhibited similar levels of viral binding, entry, and early gene expression compared to permissive subsets of 3T3 cells [60]. Similarly, the Vaccinia virus was associated equally with a fibroblast cell line and monocyte-derived dendritic cells (DCs). Nonetheless, while fibroblasts supported viral replication, the infection of DCs was abortive, activating early viral promoters but not late viral promoters [61].

In another example, human primary blood monocytes differentiated into macrophages through three different methods—using human AB serum, granulocyte–macrophage colony-stimulating factor (GM-CSF), or macrophage colony-stimulating factor (M-CSF)—all showed comparable levels of Vaccinia virus binding and early viral gene expression. However, only GM-CSF and M-CSF differentiated macrophages supported productive viral replication [62].

The ability of Poxviruses to infect human cells and express early viral genes without completing an entire infectious cycle may be advantageous for using these viruses as platforms for vaccines or genetic therapies. Our study found that both early (LSDV035 and LSDV076) and late (LSDV086, LSDV089) viral genes were transcribed in LSDV-infected PBMCs (Figures 2E-J, Additional files 8A-D). We did not observe significant differences in WT and Vaccine LSDV viral transcript levels in MDBK cells, fibroblasts, or PBMCs (Figures 2E-J, Additional file 8). Interestingly, sheep PBMCs inoculated with WT Sheeppox virus showed higher levels of viral RNA than those infected with an attenuated vaccine strain. However, it remains unclear whether either virus completed its replication cycle in PBMCs to produce infectious progeny [63]. The mechanism behind the abortive infection of PBMCs by LSDV needs further investigation.

We wanted to investigate whether the nonproductive LSDV infection of PBMCs is associated with an interferon-induced antiviral response. We found that neither the WT nor the vaccine LSDV induced the expression of ISGs in PBMCs. In contrast, ISGs were strongly induced in the PBMCs inoculated with HT viruses. This suggests that through an unknown mechanism, infectious LSDV suppresses the IFN response in PBMCs (Figure 3). It also indicates that a particular component of the inactivated viral particle is causing PBMCs to mount an IFN response.

Similarly, it was previously demonstrated that heat-treated but not infectious Vaccinia virus induced the secretion of IFNα from plasmacytoid dendritic cells [64]. Interestingly, the HT Vaccine LSDV was more potent than the HT WT virus when inducing IFIT1-3 RNAs in PBMCs. It still needs to be investigated whether this difference is due to a quantitative factor (the number of physical particles) or a qualitative factor (the composition of the particles) between the vaccine and the wild-type preparations.

Our findings suggest that vaccination with a partially inactivated LSDV vaccine strain may induce a beneficial IFN stimulatory response, which would be suppressed after immunisation with a live attenuated vaccine. Such partially inactivated vaccine formulations may be advantageous by combining IFN stimulation (driven by the dominance of inactivated particles) with the capacity to raise cell-mediated immunity (driven by residual infectivity).

Recent studies have demonstrated that infectious, but not UV-inactivated, LSDV can induce the expression of IFNꞵ RNA in MDBK cells at 36 hpi and 48 hpi, but not at earlier time points [37]. Additionally, three ISGs (*ISG54, ISG56* and *Mx1*) were found to be induced by infectious LSDV starting at 24 hpi (ca twofold), with levels increasing at 36 hpi and 48 hpi. However, in our experiments, there were no significant changes in ISG expression in MDBK cells infected with either infectious or HT LSDV (Additional file 12). This discrepancy may be attributed to differences between the field isolates investigated by Liang et al. and the isolates used in our experiments.

Viruses belonging to the Pox family are recognised for their ability to spread through direct cell-to-cell interaction [65]. To investigate the potential role of PBMCs in LSDV dissemination, we conducted co-culture experiments. Our findings showed that although infected PMBCs did not support productive replication of LSDV, they did transmit the virus to co-cultured permissive MDBK cells (Figure 4). This transmission occurred through direct cell-to-cell contact, as no viral spread was observed when a transwell physically separated the two cell types.

The exact mechanism of transmission is still under investigation. It may involve several pathways: (i) Infectious LSDV particles could interact with PBMCs from the outside without actually entering the cells. (ii) Alternatively, LSDV may be transferred after entering PBMCs, or by both of these routes. (iii) Additionally, there is the possibility of cell-to-cell transmission through replicative naked viral genomes [66].

Our findings suggest that PBMCs may serve as carriers of LSDV within the host, potentially spreading the virus to multiple permissive cells and tissues. Research has shown that human monocyte-derived macrophages that support productive Vaccinia virus replication can form structures associated with virions, which may facilitate cell-to-cell spread [62].

Moreover, white blood cells have been identified as potential virus carriers, even without supporting viral replication. For instance, the foot and mouth disease virus (FMDV), a significant pathogen affecting cattle and other livestock, was observed to be phagocytosed by macrophages and remained infectious within these cells despite not showing any viral RNA synthesis. Furthermore, FMDV-infected macrophages could transfer the virus to co-cultured permissive BHK-12 cells [67].

We have discovered that both virulent and attenuated LSDV effectively replicate in MDBK, primary fibroblasts, and BoMac cells, but they replicate poorly in primary PBMCs. However, PBMCs can still facilitate the dissemination of LSDV to permissive cells. Whether a subset of blood cells not cultured under our experimental conditions could support efficient replication of LSDV in vivo remains unknown. Our findings indicate that infectious LSDV suppresses the expression of ISGs in PBMCs. Investigating the mechanisms that limit LSDV replication in PMBCs may provide insights into the complex interactions between this virus and host cells.

## Supplementary Information

**Additional file 1. Generation of recombinant LSDV viruses expressing GFP.** (A) GFP was cloned under a Vaccinia virus synthetic promoter (MVA-pS, [40]) flanked by sequences with homology to the ends of LSDV05 and LSDV06 genes. Broad arrows in the scheme represent the orientations of viral ORFs. Thin coloured lines represent predicted amplicon sizes using the specified primer sets (detailed in Additional file 2). (B) LSDV-GFP viruses were screened for homogeneity by endpoint PCR. PCR products of DNA extracted from MDBK cells, either mock infected or infected with parental and recombinant LSDV strains, were separated by agarose gel electrophoresis using the three primer sets (A-C) illustrated above. Amplification of DNA from recombinant viruses with flanking primers gave rise to a single amplification product confirming proper integration of the EGFP cassette into the targeted LSDV genomic site with no traces of an amplicon derived from parental LSDV DNA.

**Additional file 2. List of primers used in this study.**

**Additional file 3. GFP expression in MDBK cells and primary fibroblasts infected with GFP recombinant viruses indicates viral replication.** (A-H) MDBK cells (A-D) and primary bovine fibroblasts (E-H) were inoculated with recombinant LSDV WT-GFP and LSDV Vac-GFP at MOI=3 for 1 h (T=0). Infected cells were collected and analysed by flow cytometry at the time points indicated. Histograms representing LSDV WT-GFP-infected MDBK cells (A, B) and fibroblasts cells (E, F) show GFP build-up in infected cells at the indicated time points. The percentage of GFP-expressing cells (C, MDBK cells and G, fibroblasts cells) and the mean fluorescence intensity (D, MDBK cells and H, fibroblasts cells) are presented. Values in graphs were expressed as mean± SEM representing three biological replicates (C- D and G- H).

**Additional file 4. Both non-stimulated and pokeweed mitogen-stimulated PBMCs fail to support productive LSDV replication.** (A) Non-stimulated PBMCs were inoculated with recombinant LSDV WT-GFP and LSDV VAC-GFP at MOI=3. After ~2 h of inoculation and wash (T=0) and after 48 h, cells were collected for flow cytometry analysis, and mean fluorescence intensity was calculated (accompanies Figures 2A-C). Bar graphs were plotted from five biological replicates, presented as mean± SEM. (B) PBMCs were either non-stimulated or stimulated with pokeweed mitogen by overnight incubation and then inoculated with LSDV or LSDV  GFP strains at MOI=1 for 1 h. At indicated times, cells were collected, viral DNA extracted, and relative genome copies quantified by qPCR. Two sets were used to plot a bar graph (values expressed as mean± SD). One-way ANOVA following Tukey’s post-hoc test was used to derive significance. NS- non-significant.

**Additional file 5. De-novo synthesised GFP is an assay mark of PBMCs susceptibility to LSDV infection.** (A) PBMCs were inoculated with either infectious LSDV-GFP at MOI=1 or with the same stock volume of heat-treated (HT) viruses. After 24 hpi, cells were visualised using fluorescence microscopy. The few GFP-positive cells infected with HT-GFP viruses may represent incomplete inactivation of LSDV-GFP viruses. (B) PBMCs infected with LSDV-GFP viruses at MOI=1 with or without CHX pretreatment. Images taken after 12 h (B). Representative images (A and B) from three biological repeats are presented (scale bar-100 µm). (C) Viral stocks were heat incubated for 30 min at 55 °C in a water bath. Stocks were then used to determine any loss of infectivity, which was evaluated as TCID_50_ in MDBK cells. Four stocks grown at different times were used to draw the figure, and values were presented as mean± SEM. A t-test was used to compare the significance, ∗*p* < 0.05. (D) Fluorescence intensity of preformed GFP measured by fluorometry in LSDV-GFP (heat treated or untreated) stock ascertains no impact of heat treatment on GFP fluorescence. Three repeats were followed to draw the histogram. Values are presented as mean± SEM. One-way ANOVA was used to compare all means, and post-hoc Tukey’s test was followed to test the significance level. NS- non-significant.

**Additional file 6. Infection with LSDV-GFP does not affect PBMCs viability.** PBMCs were uninfected or infected with GFP-LSDV strains at MOI = 1 for 1 h. After 2- and 48-h, cells were evaluated for viability using propidium iodide (PI) exclusion assay—values expressed as mean ±SEM representing three biological repeats. One-way ANOVA was used to compare all means, and post-hoc Tukey’s test was followed to test the significance level. NS- non-significant.

**Additional file 7. LSDV084 (late gene) expression is affected by inhibition of viral DNA replication, in contrast to LSDV035 (early gene).** (A-B) MDBK cells, either untreated or treated with PAA, were infected with LSDV at MOI=1 for 1h and checked for inhibition of viral genome replication (A) and viral gene expression (B) at the indicated time points. Three biological sets were used to draw the graphs, presented as mean± SEM. Paired t-test (A) and one-way ANOVA followed by post-hoc Tukey’s test were used to test significance. *p < 0.05.

**Additional file 8. LSDV genes follow a similar trend of expression in MDBK cells, PBMCs and fibroblasts.** (A-D) LSDV076 and LSDV089 expression levels in MDBK cells and PBMCs infected with LSDV- GFP strains (Supplementary data Figure 2E-J). Three repeats were plotted in the bar graph as mean ± SEM. One-way ANOVA followed by post-hoc Tukey’s test was used to test significance. ** *p* < 0.01, *** *p* < 0.001, NS- non-significant. (E-I) Bovine foreskin fibroblast cells were infected at MOI-1 with either of the LSDV-GFP strains for 1 h. Relative changes in the levels of transcripts encoding the reporter gene GFP and viral genes at the time points indicated were determined by RT-qPCR. Three repeats were plotted in the bar graph as mean± SEM.

**Additional file 9. Heat treatment of LSDV reduces the expression of viral genes in infected MDBK cells.** (A-C) MDBK cells were inoculated with infectious LSDV WT-GFP at MOI=1 or with the same volume of heat-treated LSDV for 1 h. Relative changes in the levels of transcripts encoding the reporter gene GFP and viral genes (LSDV035 and LSDV84) at the time points indicated were determined by RT-qPCR. Values were presented as mean± SEM.

**Additional file 10. Heat treatment of LSDV reduces the expression of viral genes in inoculated PBMCs.** (A-C) PBMCs were inoculated for 1 h with infectious LSDV WT-GFP at MOI=1 or with the same LSDV dose that was heat-treated LSDV before inoculation. Relative changes in the levels of transcripts encoding the reporter gene GFP and viral genes (LSDV035 and LSDV84) at the time points indicated were determined by RT-qPCR. One-way ANOVA with the Sidak multi-comparison test was used to measure the significance level. * *p* < 0.05, ** *p* < 0.01 and ***p < 0.001.

**Additional file 11. LSDV transcripts’ levels are only mildly affected by CHX treatment in infected PBMCs.** (A-C) PBMCs either untreated or treated with CHX were infected with LSDV WT-GFP at MOI=1 for 1h. Relative changes in the levels of transcripts encoding the reporter gene GFP and viral genes (LSDV035 and LSDV84) at the time points indicated were determined by RT-qPCR. Values are shown as mean ± SEM of three biological repeats. One-way ANOVA followed by post-hoc Tukey’s test was used to test significance. NS- non-significant.

**Additional file 12. MDBK and BoMac cells infected with LSDV show insignificant changes in ISGs’ RNA levels.** (A-C) MDBK or BoMac cells (D-F) were infected with LSDV-GFP viruses at MOI=1 for 1h. Relative changes in the levels of IFI1-3 transcripts at the time points indicated were determined by RT-qPCR. RNA used in (A-C) were from the experiment described in Figures 2E-J. Values are shown as mean ± SEM of three biological repeats.

**Additional file 13. LSDV GFP viruses disseminated to permissive cells from infected PBMCs by direct contact.** PBMCs (A) or primary fibroblasts (B) infected with LSDV-GFP strains were co-cultured with MDBK-Ruby cells constitutively expressing the red fluorescent protein mRuby3. Images were acquired at 4 dpi (A) or at the indicated times (B). White arrowheads depict infected fibroblasts (GFP-positive, Ruby negative), which disseminate at 2 dpi to MDBK-Ruby cells (white stars, double positive for GFP and Ruby). (C) To evaluate the contribution of indirect versus direct LSDV-GFP transmission from infected PBMCs, MDBK cells were either seeded into a transwell inset (0.4 µ membrane pore size) placed into a well containing infected PBMCs (C, left) or directly overlaid on the infected PBMCs (C, right). At 4 dpi, cells were fixed, counterstained with Hoechst (DNA stain) and imaged to reveal GFP-positive cells infected by LSDV-GFP. Scale bar 100 µm (A, B), 500 µm (C).

## Data Availability

The datasets during and/or analysed during the current study are available from the corresponding author upon reasonable request.
