# Correction: Lumpy skin disease virus suppresses the antiviral response of bovine peripheral blood mononuclear cells that support viral dissemination

**DOI:** 10.1186/s13567-025-01544-6

**Published:** 2025-06-12

**Authors:** Manoj Kumar, Ohad Frid, Asaf Sol, Alexander Rouvinski, Sharon Karniely

**Affiliations:** 1 Department of Microbiology and Molecular Genetics, The Hebrew University‑Hadassah Medical School, Institute for Medical Research Israel‑Canada, The Hebrew University of Jerusalem, Jerusalem, Israel; 2 Department of Virology, Kimron Veterinary Institute, Beit Dagan, Israel; 3 Department of Parasitology, Kimron Veterinary Institute, Beit Dagan, Israel; 4 The Kuvin Center for the Study of Infectious and Tropical Diseases, The Hebrew University Hadassah Medical School, The Hebrew University of Jerusalem, Jerusalem, Israel

**Correction: Veterinary Research (2025) 56:93** 10.1186/s13567-025-01516-w

Following publication of the original article [1], the authors identified an error in Figure 1 and Figure 2:

1. Main Figure 1: **A** was added to mark the first panel, the label of the lowest panel of images in A was corrected to LSDV-Vac-GFP.
2. Main Figure 2: Labels in panels H and I were corrected to LSDV Vac-GFP and LSDV Vac.

The original article has been corrected.
