# Supplementary figures and images for: Lumpy skin disease virus suppresses the antiviral response of bovine peripheral blood mononuclear cells that support viral dissemination
Source: Vet Res. 2025 Apr 26;56:93. doi: 10.1186/s13567-025-01516-w (PMC12034137; doi:10.1186/s13567-025-01516-w)

**A**

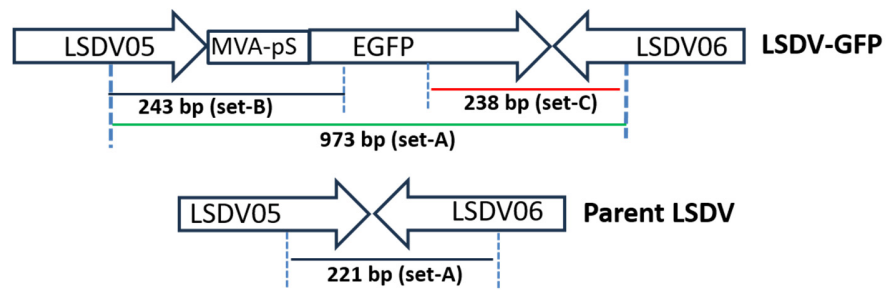

**B**

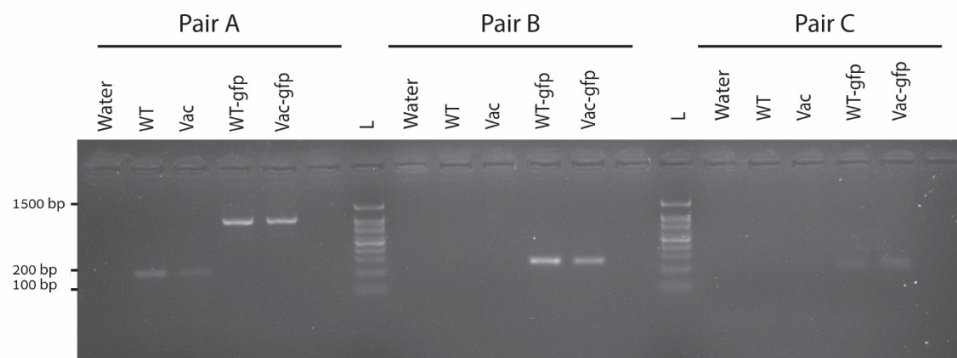

Supplement: Supplementary file 1 — Additional file 1. Generation of recombinant LSDV viruses expressing GFP. (A) GFP was cloned under a Vaccinia virus synthetic promoter (MVA-pS, [40]) flanked by sequences with homology to the ends of LSDV05 and LSDV06 genes. Broad arrows in the scheme represent the orientations of viral ORFs. Thin coloured lines represent predicted amplicon sizes using the specified primer sets (detailed in Additional file 2). (B) LSDV-GFP viruses were screened for homogeneity by endpoint PCR. PCR products of DNA extracted from MDBK cells, either mock infected or infected with parental and recombinant LSDV strains, were separated by agarose gel electrophoresis using the three primer sets (A-C) illustrated above. Amplification of DNA from recombinant viruses with flanking primers gave rise to a single amplification product confirming proper integration of the EGFP cassette into the targeted LSDV genomic site with no traces of an amplicon derived from parental LSDV DNA. [file 13567_2025_1516_MOESM1_ESM.pdf]

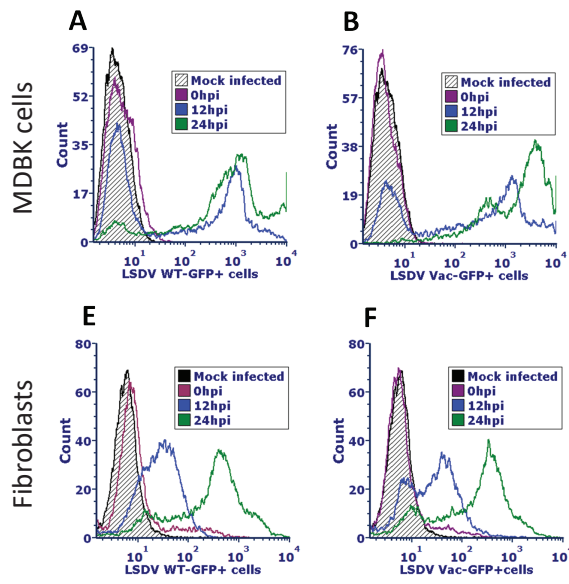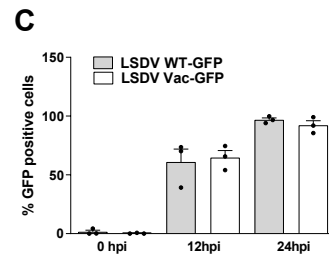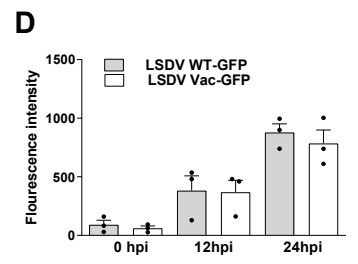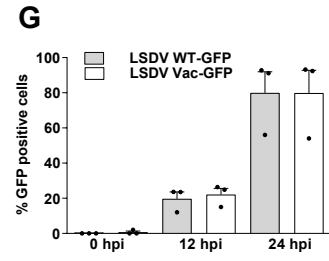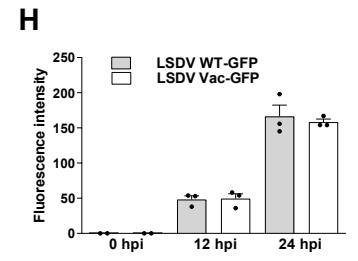

Supplement: Supplementary file 3 — Additional file 3. GFP expression in MDBK cells and primary fibroblasts infected with GFP recombinant viruses indicates viral replication. (A-H) MDBK cells (A-D) and primary bovine fibroblasts (E-H) were inoculated with recombinant LSDV WT-GFP and LSDV Vac-GFP at MOI=3 for 1 h (T=0). Infected cells were collected and analysed by flow cytometry at the time points indicated. Histograms representing LSDV WT-GFP-infected MDBK cells (A, B) and fibroblasts cells (E, F) show GFP build-up in infected cells at the indicated time points. The percentage of GFP-expressing cells (C, MDBK cells and G, fibroblasts cells) and the mean fluorescence intensity (D, MDBK cells and H, fibroblasts cells) are presented. Values in graphs were expressed as mean± SEM representing three biological replicates (C- D and G- H). [file 13567_2025_1516_MOESM3_ESM.pdf]

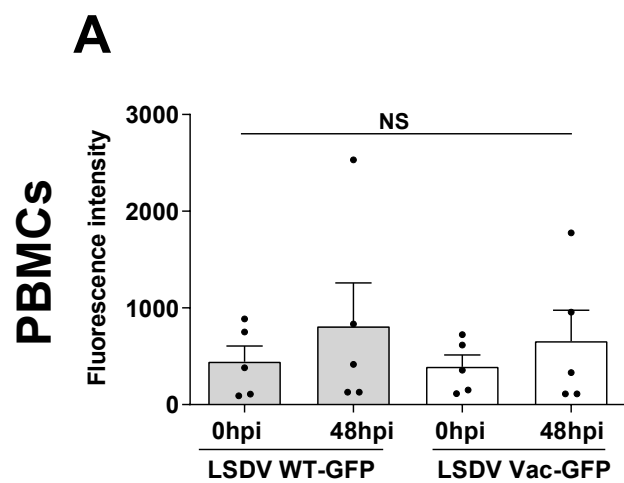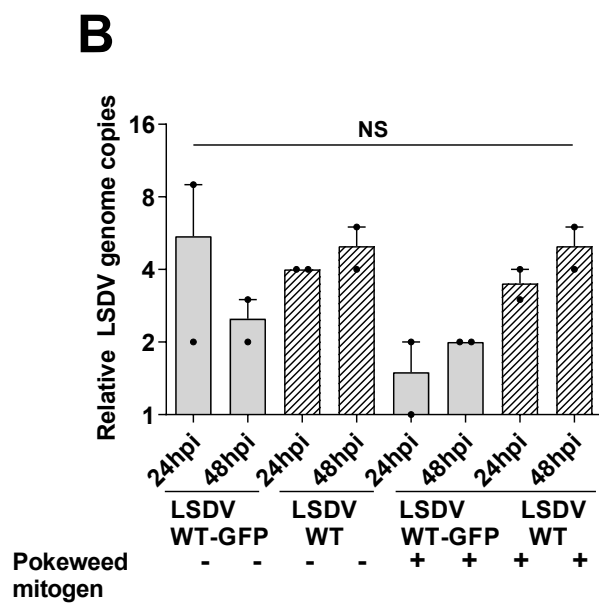

Supplement: Supplementary file 4 — Additional file 4. Both non-stimulated and pokeweed mitogen-stimulated PBMCs fail to support productive LSDV replication. (A) Non-stimulated PBMCs were inoculated with recombinant LSDV WT-GFP and LSDV VAC-GFP at MOI=3. After ~2 h of inoculation and wash (T=0) and after 48 h, cells were collected for flow cytometry analysis, and mean fluorescence intensity was calculated (accompanies Figures 2A-C). Bar graphs were plotted from five biological replicates, presented as mean± SEM. (B) PBMCs were either non-stimulated or stimulated with pokeweed mitogen by overnight incubation and then inoculated with LSDV or LSDV GFP strains at MOI=1 for 1 h. At indicated times, cells were collected, viral DNA extracted, and relative genome copies quantified by qPCR. Two sets were used to plot a bar graph (values expressed as mean± SD). One-way ANOVA following Tukey’s post-hoc test was used to derive significance. NS- non-significant. [file 13567_2025_1516_MOESM4_ESM.pdf]

**A**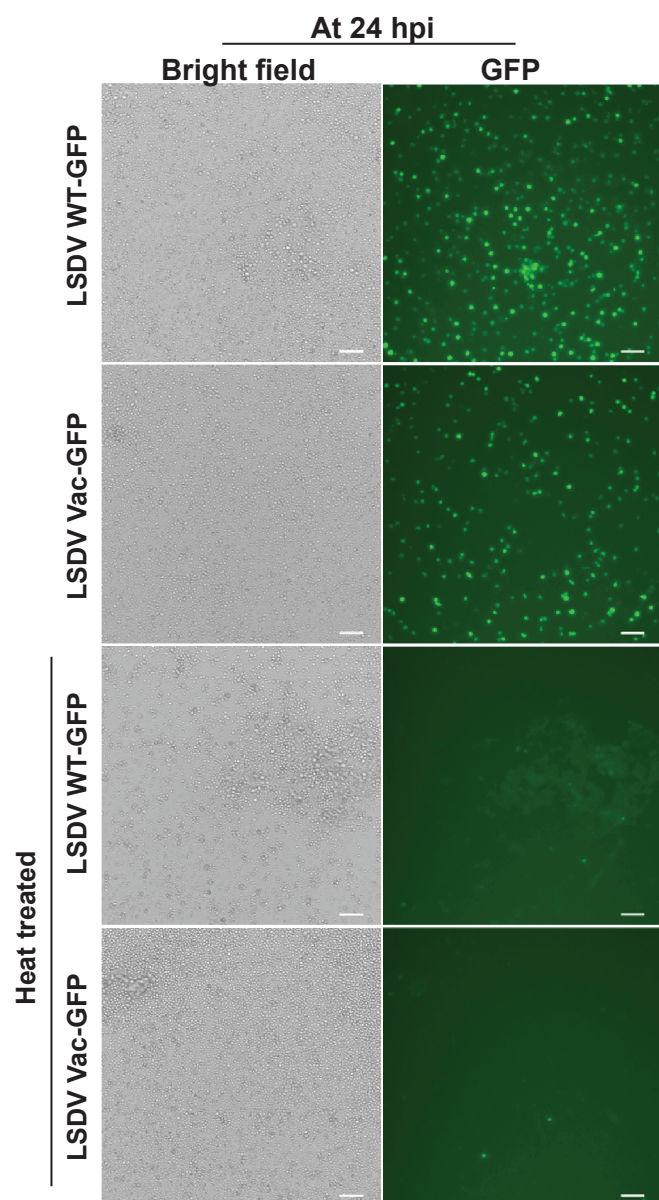**B**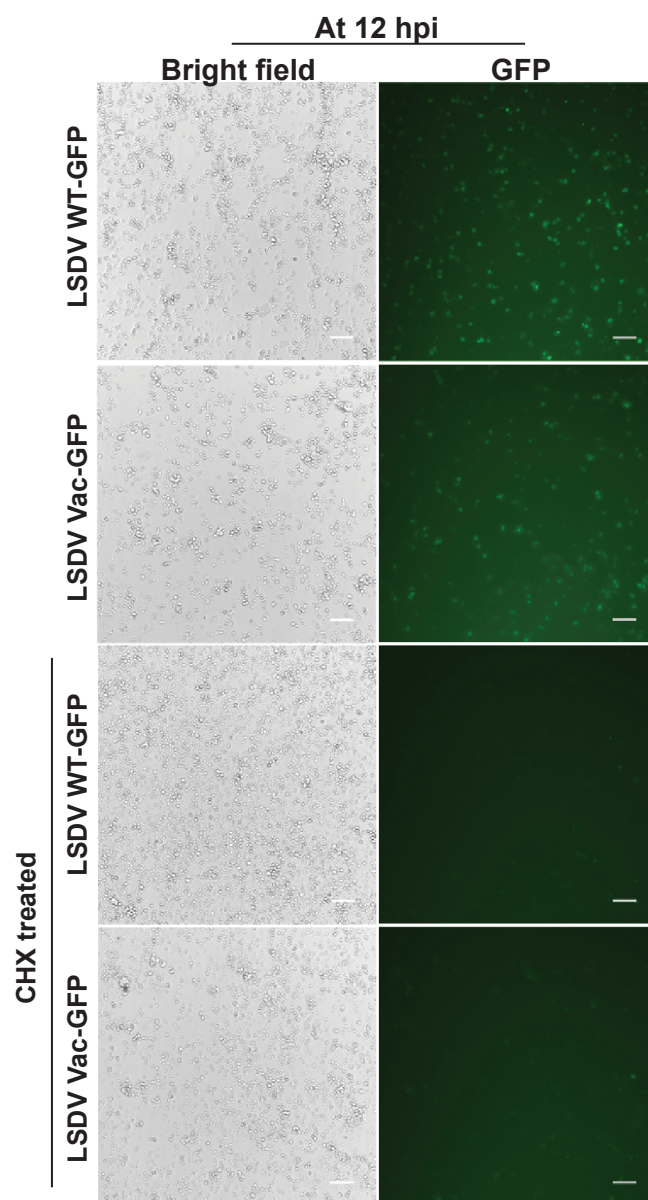**C**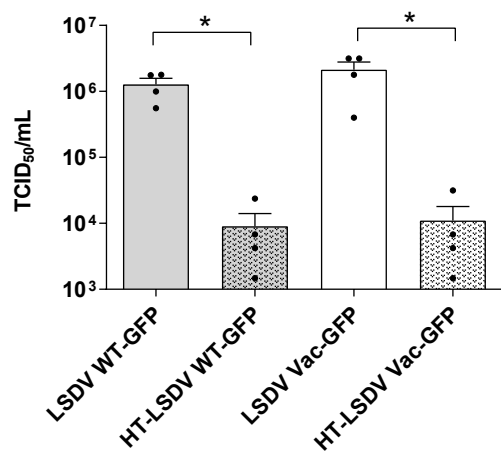**D**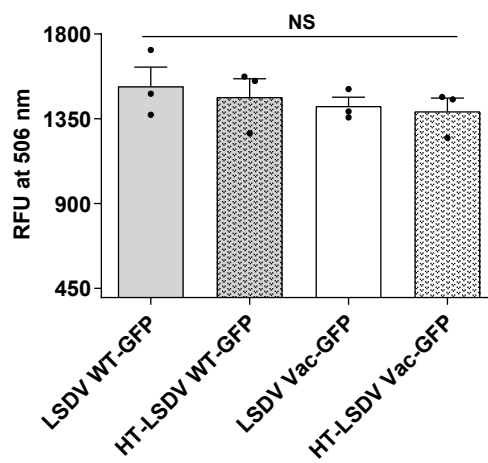

Supplement: Supplementary file 5 — Additional file 5. De-novo synthesised GFP is an assay mark of PBMCs susceptibility to LSDV infection. (A) PBMCs were inoculated with either infectious LSDV-GFP at MOI=1 or with the same stock volume of heat-treated (HT) viruses. After 24 hpi, cells were visualised using fluorescence microscopy. The few GFP-positive cells infected with HT-GFP viruses may represent incomplete inactivation of LSDV-GFP viruses. (B) PBMCs infected with LSDV-GFP viruses at MOI=1 with or without CHX pretreatment. Images taken after 12 h (B). Representative images (A and B) from three biological repeats are presented (scale bar-100 µm). (C) Viral stocks were heat incubated for 30 min at 55 °C in a water bath. Stocks were then used to determine any loss of infectivity, which was evaluated as TCID50 in MDBK cells. Four stocks grown at different times were used to draw the figure, and values were presented as mean± SEM. A t-test was used to compare the significance, ∗p < 0.05. (D) Fluorescence intensity of preformed GFP measured by fluorometry in LSDV-GFP (heat treated or untreated) stock ascertains no impact of heat treatment on GFP fluorescence. Three repeats were followed to draw the histogram. Values are presented as mean± SEM. One-way ANOVA was used to compare all means, and post-hoc Tukey’s test was followed to test the significance level. NS- non-significant. [file 13567_2025_1516_MOESM5_ESM.pdf]

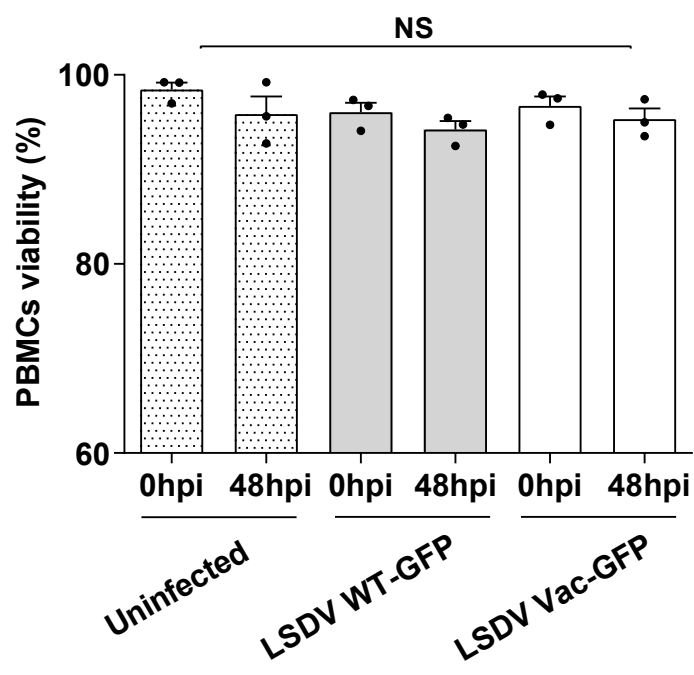

Supplement: Supplementary file 6 — Additional file 6. Infection with LSDV-GFP does not affect PBMCs viability. PBMCs were uninfected or infected with GFP-LSDV strains at MOI = 1 for 1 h. After 2- and 48-h, cells were evaluated for viability using propidium iodide (PI) exclusion assay—values expressed as mean ±SEM representing three biological repeats. One-way ANOVA was used to compare all means, and post-hoc Tukey’s test was followed to test the significance level. NS- non-significant. [file 13567_2025_1516_MOESM6_ESM.pdf]

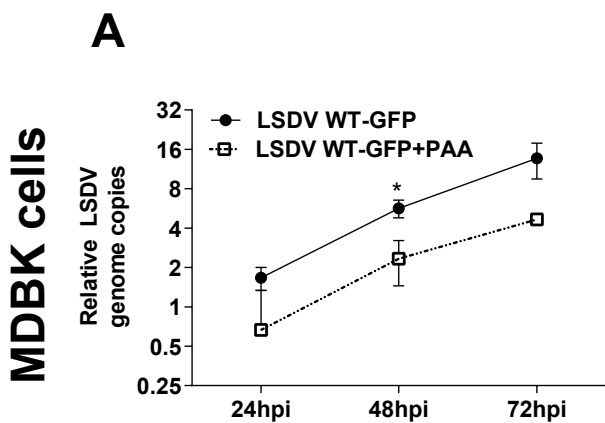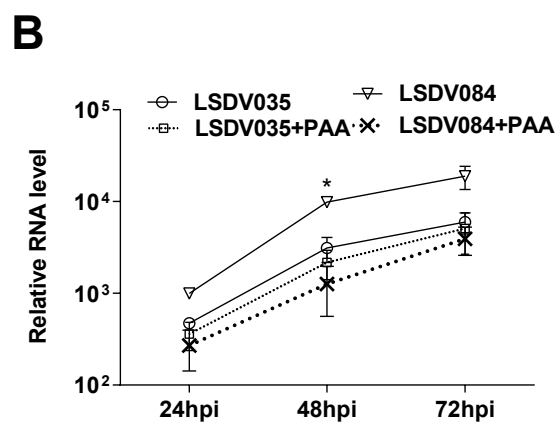

Supplement: Supplementary file 7 — Additional file 7. LSDV084 (late gene) expression is affected by inhibition of viral DNA replication, in contrast to LSDV035 (early gene). (A-B) MDBK cells, either untreated or treated with PAA, were infected with LSDV at MOI=1 for 1h and checked for inhibition of viral genome replication (A) and viral gene expression (B) at the indicated time points. Three biological sets were used to draw the graphs, presented as mean± SEM. Paired t-test (A) and one-way ANOVA followed by post-hoc Tukey’s test were used to test significance. *p < 0.05. [file 13567_2025_1516_MOESM7_ESM.pdf]

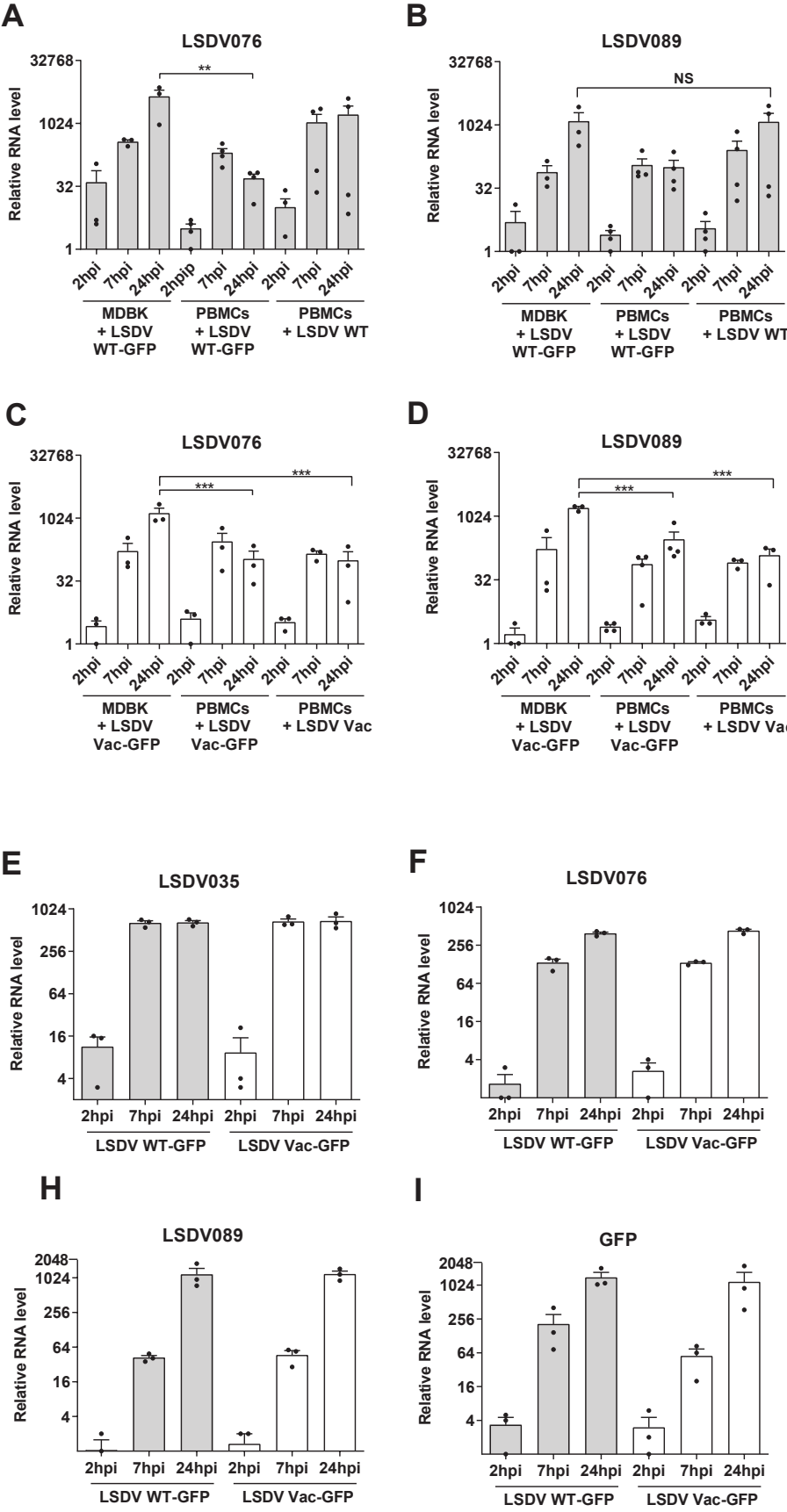

Supplement: Supplementary file 8 — Additional file 8. LSDV genes follow a similar trend of expression in MDBK cells, PBMCs and fibroblasts. (A-D) LSDV076 and LSDV089 expression levels in MDBK cells and PBMCs infected with LSDV- GFP strains (Supplementary data Figure 2E-J). Three repeats were plotted in the bar graph as mean ± SEM. One-way ANOVA followed by post-hoc Tukey’s test was used to test significance. ** p < 0.01, *** p < 0.001, NS- non-significant. (E-I) Bovine foreskin fibroblast cells were infected at MOI-1 with either of the LSDV-GFP strains for 1 h. Relative changes in the levels of transcripts encoding the reporter gene GFP and viral genes at the time points indicated were determined by RT-qPCR. Three repeats were plotted in the bar graph as mean± SEM. [file 13567_2025_1516_MOESM8_ESM.pdf]

**A**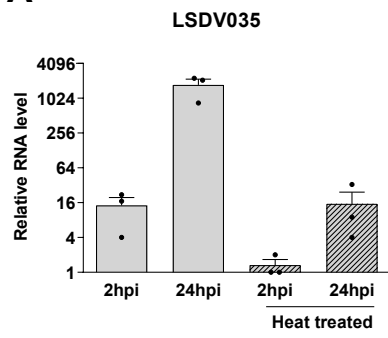**B**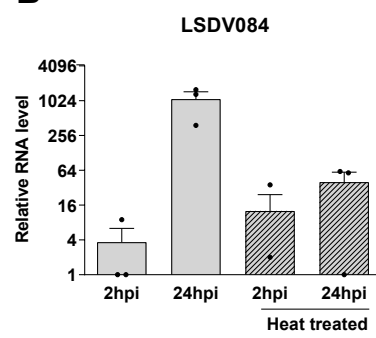**C**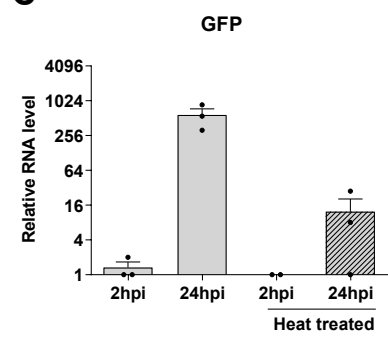

Supplement: Supplementary file 9 — Additional file 9. Heat treatment of LSDV reduces the expression of viral genes in infected MDBK cells. (A-C) MDBK cells were inoculated with infectious LSDV WT-GFP at MOI=1 or with the same volume of heat-treated LSDV for 1 h. Relative changes in the levels of transcripts encoding the reporter gene GFP and viral genes (LSDV035 and LSDV84) at the time points indicated were determined by RT-qPCR. Values were presented as mean± SEM. [file 13567_2025_1516_MOESM9_ESM.pdf]

**A**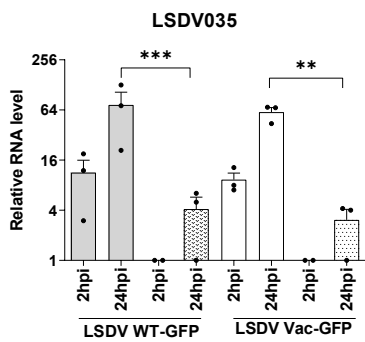

Heat treated

**B**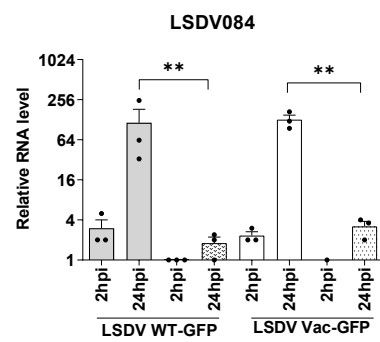

Heat treated

**C**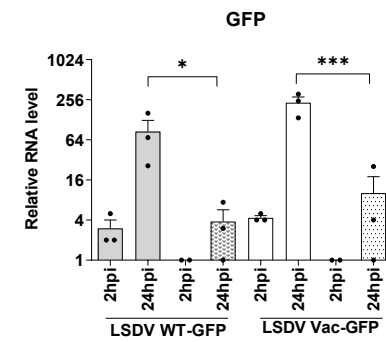

Heat treated

Supplement: Supplementary file 10 — Additional file 10. Heat treatment of LSDV reduces the expression of viral genes in inoculated PBMCs. (A-C) PBMCs were inoculated for 1 h with infectious LSDV WT-GFP at MOI=1 or with the same LSDV dose that was heat-treated LSDV before inoculation. Relative changes in the levels of transcripts encoding the reporter gene GFP and viral genes (LSDV035 and LSDV84) at the time points indicated were determined by RT-qPCR. One-way ANOVA with the Sidak multi-comparison test was used to measure the significance level. * p < 0.05, ** p < 0.01 and ***p < 0.001. [file 13567_2025_1516_MOESM10_ESM.pdf]

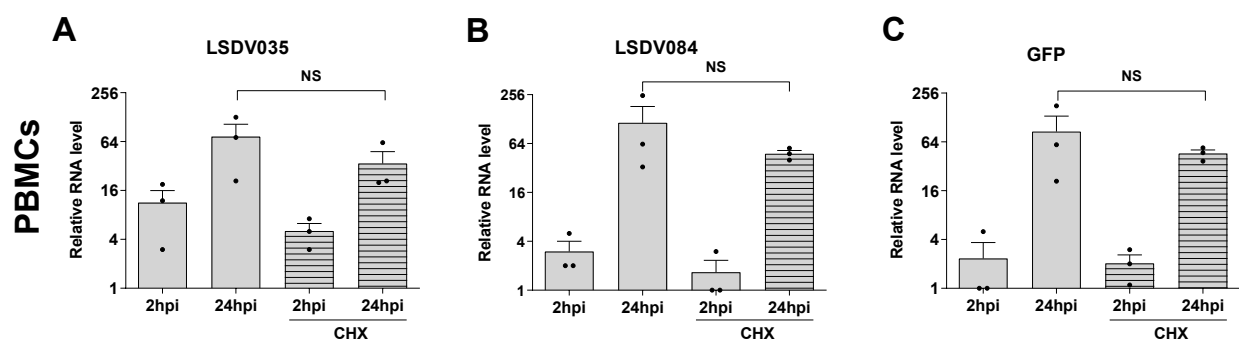

Supplement: Supplementary file 11 — Additional file 11. LSDV transcripts’ levels are only mildly affected by CHX treatment in infected PBMCs. (A-C) PBMCs either untreated or treated with CHX were infected with LSDV WT-GFP at MOI=1 for 1h. Relative changes in the levels of transcripts encoding the reporter gene GFP and viral genes (LSDV035 and LSDV84) at the time points indicated were determined by RT-qPCR. Values are shown as mean ± SEM of three biological repeats. One-way ANOVA followed by post-hoc Tukey’s test was used to test significance. NS- non-significant. [file 13567_2025_1516_MOESM11_ESM.pdf]

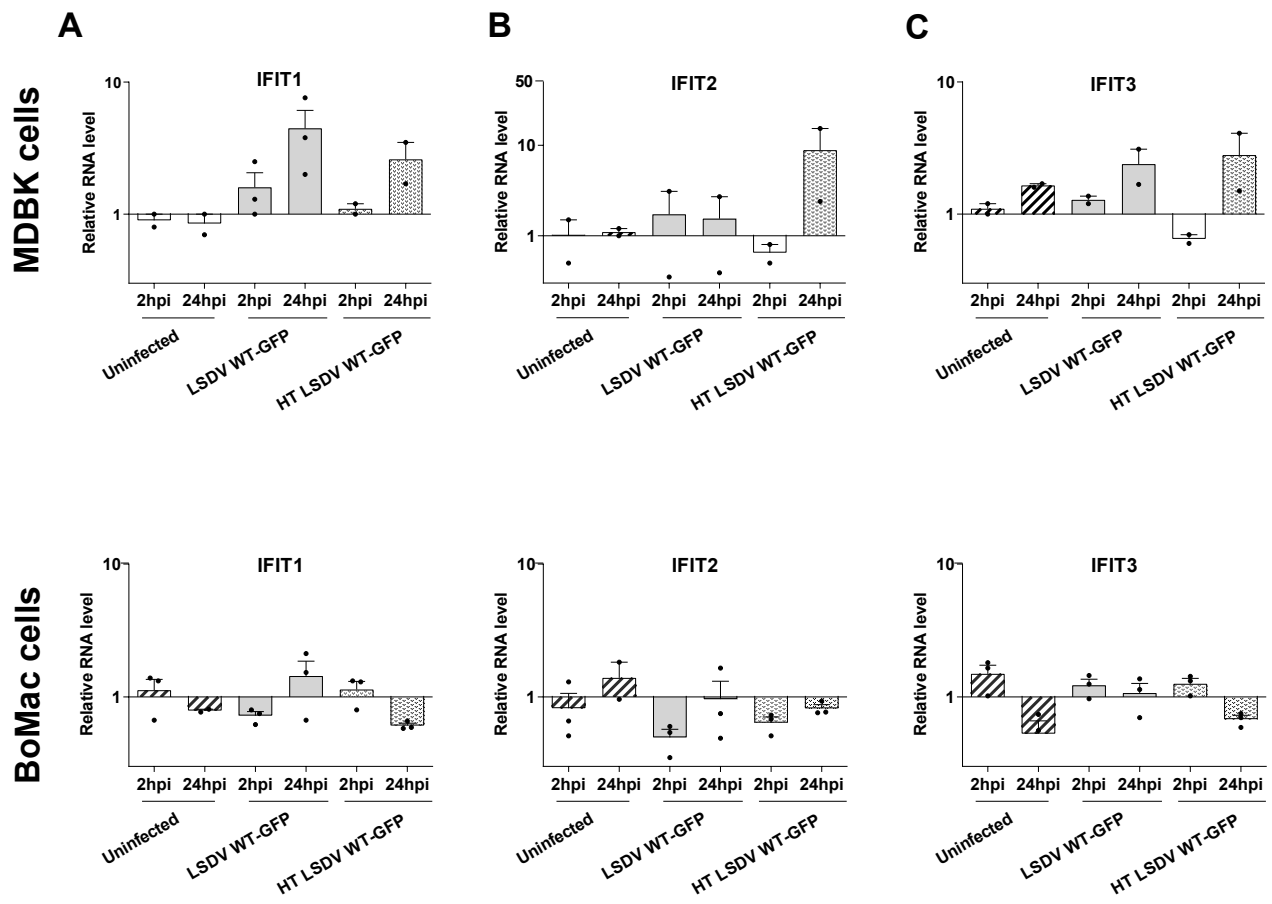

Supplement: Supplementary file 12 — Additional file 12. MDBK and BoMac cells infected with LSDV show insignificant changes in ISGs’ RNA levels. (A-C) MDBK or BoMac cells (D-F) were infected with LSDV-GFP viruses at MOI=1 for 1h. Relative changes in the levels of IFI1-3 transcripts at the time points indicated were determined by RT-qPCR. RNA used in (A-C) were from the experiment described in Figures 2E-J. Values are shown as mean ± SEM of three biological repeats. [file 13567_2025_1516_MOESM12_ESM.pdf]

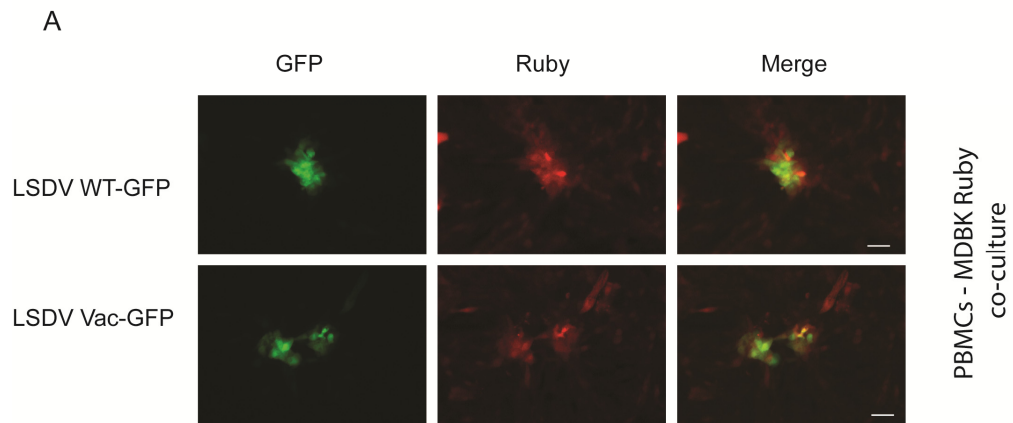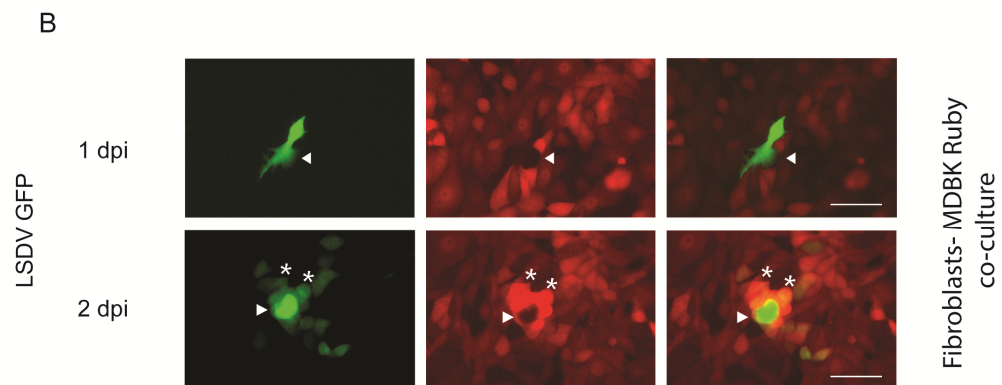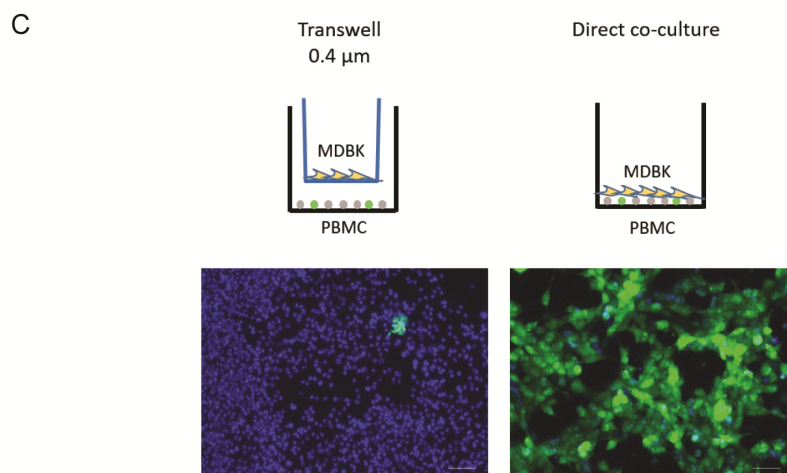

Supplement: Supplementary file 13 — Additional file 13. LSDV GFP viruses disseminated to permissive cells from infected PBMCs by direct contact. PBMCs (A) or primary fibroblasts (B) infected with LSDV-GFP strains were co-cultured with MDBK-Ruby cells constitutively expressing the red fluorescent protein mRuby3. Images were acquired at 4 dpi (A) or at the indicated times (B). White arrowheads depict infected fibroblasts (GFP-positive, Ruby negative), which disseminate at 2 dpi to MDBK-Ruby cells (white stars, double positive for GFP and Ruby). (C) To evaluate the contribution of indirect versus direct LSDV-GFP transmission from infected PBMCs, MDBK cells were either seeded into a transwell inset (0.4 µ membrane pore size) placed into a well containing infected PBMCs (C, left) or directly overlaid on the infected PBMCs (C, right). At 4 dpi, cells were fixed, counterstained with Hoechst (DNA stain) and imaged to reveal GFP-positive cells infected by LSDV-GFP. Scale bar 100 µm (A, B), 500 µm (C). [file 13567_2025_1516_MOESM13_ESM.pdf]
