# Supplementary material for: Lumpy skin disease virus suppresses the antiviral response of bovine peripheral blood mononuclear cells that support viral dissemination
Source: Vet Res. 2025 Apr 26;56:93. doi: 10.1186/s13567-025-01516-w (PMC12034137; doi:10.1186/s13567-025-01516-w)
Supplement: Supplementary file 2 — Additional file 2. List of primers used in this study. [file 13567_2025_1516_MOESM2_ESM.pdf]

Primers list

#	Primer sets	Sequence (5'-3')	Amplicon (bp)	Notes/Remarks/Gene ID
1.	LSDV035F	CCATCTCGCGCTAGATGAATAC	149	27% homology to VV gene (E5R) under an early promoter
	LSDV035R	TGTATCCTAGCTTTTTCGGAGG		
2.	LSDV076F	AGGTAGCGGACGAAATCTTTCC	93	37 % homology to VV gene-H5R under an early promoter
	LSDV076R	CCGCTTTCTTACGAACAACTGG		
3.	LSDV084F	AATGGGTCACAAGGGACAAATC	137	81% homology to VV gene-D6R under a late promoter
	LSDV084R	TGGCTCCCATCATCATTTGTTG		
4.	LSDV089F	TTGGACATTGTTGGCATCCTTC	115	74% homology to VV gene-D12L under E/L promoter
	LSDV089R	AGATAGTGCGATTATGGTAGCG		
5.	GFP-F	CACCATCTTCTTCAAGGACGAC	212	Expressed under vaccinia synthetic early/late promoter
	GFP-R	TCTTGAAGTTCACCTTGATGCC		
6.	GAPDH-F(RNA)	AGGTCGGAGTGAACGGATTC	197	Self-design, NM_001034034.2
	GAPDH-R(RNA)	CATTGATGACGAGCTTCCCG		
7.	GAPDH-F(DNA)	GTGATGCTGGTGCTGAGTAT	139	NC_037332, (Wang et al., 2017)
	GAPDH-R(DNA)	GCTCTCACATTCCTAAGTCC		
8.	CAPRI-F	AAATGAAACCAATGGATGGGATA	89	(Bowden et al., 2008)
	CAPRI-R	AAAACGGTATATGGAATAGAGTT		
9.	LSDV05-F	AAGGCTATGGGAGAGTTTG	973 (Pair-A)	Self-design, LSDV005 and LSDV006
	LSDV06-R	CGATGATAGGATACAGAG		
10.	EGFP-F-reco	AACGAGAAGCGCGATCAC	243 (Pair-B)	Self-design, pEGFP-N1 and LSDV006
	LSDV06-R	CGATGATAGGATACAGAG		
11.	LSDV05-F	AAGGCTATGGGAGAGTTTG	238 (Pair-C)	Self-design, LSDV005 And pEGFP-N1
	EGFP-R-reco	GATGAACTTCAGGGTCAGC		
12.	LSDV05-F1	**GCTCCCGGCCGCCATGG*CCGCGG*** GATATGAAAACAAACACAAAAATAATAC		Self-design, LSDV005, (Note- letters in italics show restriction digestion sites, bold letters represent pGEMT terminal sequence and bold underline showing vaccinia derived promoter sequence)
	LSDV05-R1	**<u>TATTTATATTCCAAAAAAAAAAAATAAAATTTCAATTTT</u>***GAATTC*TTACTTTTTAACTATGTACTTTTC		

13.	EGFP-F	AGTACATAGTTAAAAAGTAAAAAATTGAAATTTTATTTTTTTTTTTGGAATATAAATAATGGTGAGC AAGGGCGAG		Self-design, pEGFP-N1
	EGFP-R	TTGTAGTTTTATAA*CTCGAG*TTACTTGTACAGCTCGTCCATGC		
14.	LSDV06-F2	*CTCGAG*TTATAAAACTACAACATTATAG		Self-design, LSDV006 (note- letters in italics show restriction digestion site and bold letters represent plasmid terminal sequence)
	LSDV06-R2	**CAGGCGGCCGCACTAGTG***ACTAGT*AAATGAAAGTTATAAATTTTATC		
15.	IFIT1-F	GGAACGTGCTGTGCAACTAA	136	XM_010819765.1, (Cheng et al., 2017)
	IFIT1-R	TTTGTCGAGTGCTTTCATGC		
16.	IFIT2-F	ACCCCATTAACCCTTTGAGG	249	BT025389.1, (Stepke et al., 2018)
	IFIT2-R	TGTTGGGCATGCATTTTAGA		
17.	IFIT3-F	TGCTGACAAGGTGAAACGAG	111	NM_001075414, (Cheng et al., 2017)
	IFIT3-R	TTTTTCCCACCGCACTTTAC		
18.	IFITM3-F	GTGGCATTCGCCTACTCTGT	142	NM_001078141.2, (Stepke et al., 2018)
	IFITM3-R	CGATGAGGACGACAGTCAGA		
20.	ISG15-F	AGAAGATCAATGTGCCTGCTTT	161	NM_174366.1, (Cheng et al., 2017)
	ISG15-R	CTTGTCGTTCCTCACCAGGAT		
21.	CXCLlO-F	ACACCGAGGCACTACGTTCT	115	NM_001046551, (Rusk et al., 2017)
	CXCLl0-R	TAAGCCCAGAGCTGGAAAGA		

[1] Bowden T.R., Babiuk S.L., Parkyn G.R., Copps J.S., Boyle D.B. (2008) Capripoxvirus tissue tropism and shedding: A quantitative study in experimentally infected sheep and goats. Virology 371:380-393

[2] Cheng Z., Chauhan L., Barry A.T., Abudureyimu A., Oguejiofor C.F., Chen X., Wathes D.C. (2017) Acute bovine viral diarrhea virus infection inhibits expression of interferon tau-stimulated genes in bovine endometrium. Biol Reprod 96:1142-1153

[3] Stepke C., Fredericksen F., Arriagada V., Oltra J., Toledo C., Villalba M., Olavarría V.J.J.M.I. (2018) Immune gene expression during bovine Herpesvirus-1 infection of MDBK cells: molecular characterization of interferon-stimulated gene 15. 3:2

[4] Wang J., Xiang H., Liu L., Kong M., Yin T., Zhao X. (2017) Mitochondrial haplotypes influence metabolic traits across bovine inter- and intra-species cybrids. Sci Rep 7:4179
